# Vps501, a novel vacuolar SNX‐BAR protein cooperates with the SEA complex to regulate TORC1 signaling

**DOI:** 10.1111/tra.12833

**Published:** 2022-02-15

**Authors:** Shreya Goyal, Verónica A. Segarra, Nitika, Aaron M. Stecher, Andrew W. Truman, Adam M. Reitzel, Richard J. Chi

**Affiliations:** ^1^ Department of Biological Sciences University of North Carolina Charlotte North Carolina USA; ^2^ Department of Biology High Point University High Point North Carolina USA

**Keywords:** Atg27, autophagy, retromer, SEA complex, SNX‐BAR, TORC1

## Abstract

The sorting nexins (SNXs), constitute a diverse family of molecules that play varied roles in membrane trafficking, cell signaling, membrane remodeling, organelle motility and autophagy. In particular, the SNX‐Bin‐Amphiphysin‐Rvs (BAR) proteins, a SNX subfamily characterized by a C‐terminal dimeric BAR lipid curvature domain and a conserved Phox‐homology domain, are of great interest. In budding yeast, many SNX‐BAR proteins have well‐characterized endo‐vacuolar trafficking roles. Phylogenetic analyses allowed us to identify an additional SNX‐BAR protein, Vps501, with a novel endo‐vacuolar role. We report that Vps501 uniquely localizes to the vacuolar membrane and has physical and genetic interactions with the SEA complex to regulate TORC1 inactivation. We found cells displayed a severe deficiency in starvation‐induced/nonselective autophagy only when SEA complex subunits are ablated in combination with Vps501, indicating a cooperative role with the SEA complex during TORC1 signaling during autophagy induction. Additionally, we found the SEACIT complex becomes destabilized in *vps501*Δ*sea1*Δ cells, which resulted in aberrant endosomal TORC1 activity and subsequent Atg13 hyperphosphorylation. We have also discovered that the vacuolar localization of Vps501 is dependent upon a direct interaction with Sea1 and a unique lipid binding specificity that is also required for its function.

## Graphical abstract

Phylogenetic analyses allowed us to identify a novel yeast SNX‐BAR protein, we have named Vps501. Here we report that Vps501 uniquely localizes to the vacuolar membrane and has physical and genetic interactions with the SEA complex to regulate TORC1. Our results indicate Vps501 acts as a stabilizer for the SEA complex to the vacuolar membrane and promotes SEACIT inactivation of vacuole and endosomal pools of TORC1.

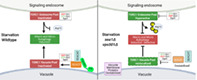

Vps501 stabilizes the SEA complex to the vacuolar membrane and promotes SEACIT inactivation.

## 1 INTRODUCTION

The sorting nexin (SNX) family is an evolutionarily conserved class of cellular trafficking proteins that are most well known for their ability to bind phospholipids to catalyze endosomal sorting reactions and other membrane trafficking pathways in the cell.^1^, ^2^ SNX proteins are structurally characterized by an evolutionarily conserved region known as the Phox (PX) homology domain, which allows them to recognize the lipid composition of the endosome, most notably phosphatidylinositol‐3‐phosphate (PI3P).^3^ While all PX domains have similar core folds, consisting of three antiparallel β‐strands and three α‐helices that extend a canonical PI3P binding motif ψPxxPxK (ψ = large aliphatic amino acid), outside this region, they have relatively low sequence similarity. A recent analysis of over 39 PX domains identified a secondary His/Tyr—containing binding motif present in a subset of PX domain proteins which may explain the lipid promiscuity of some SNXs.^4^ However, the range of different phosphoinositides that associate with PX domains, and the significance of these interactions for cellular localization are still unclear. Moreover, SNX proteins are divided into subfamilies according to the presence of other characteristic domains such as a Bin‐Amphiphysin‐Rvs (BAR) domain.^5^ BAR domains allow members of the SNX‐BAR subfamily to bind high positive curvature structures, driving the formation of endosomal tubules, while also conferring the ability to form sorting complexes that facilitate cargo selection.^5^, ^6^, ^7^ The *Saccharomyces cerevisiae* genome encodes seven annotated SNX‐BAR proteins, while the human genome encodes 12.^3^, ^5^, ^8^ In addition to working with complexes such as retromer to mediate retrograde and recycling trafficking of cargos at the tubular endosomal network,^3^, ^5^, ^6^, ^7^, ^9^ SNX‐BAR proteins contribute to other important conserved cellular processes such as macroautophagy (herein referred to as autophagy) and selective autophagy.^10^, ^11^, ^12^

Autophagy is a stress response in which eukaryotic cells recycle damaged or unneeded components by sequestering them in double‐bilayered compartments called autophagosomes. Once made, autophagosomes deliver their contents for breakdown by docking and fusing with the cell's degradative organelle, the lysosome in animal cells or the vacuole in plant and yeast cells. These key steps and the core autophagy‐related (Atg) proteins that mediate and regulate them are evolutionarily conserved across all autophagy pathways, including starvation‐induced bulk autophagy and cargo‐selective autophagy pathways.^13^, ^14^, ^15^, ^16^ While vacuoles and lysosomes can serve as storage and/or recycling depots for cells, their delimiting membranes host critical signaling events for autophagy induction such as the inactivation of *target of rapamycin* (TOR).

TORC1 has been shown to mediate multiple biological pathways that relay nutrient availability information to ensure cellular homeostasis in eukaryotic cells.^17^, ^18^, ^19^ The primary function of TORC1 is to control TOR kinase activity, linking changes in nutrient levels with transcriptomic reprogramming via its downstream effectors to promote cell proliferation.^20^ In yeast, TORC1 has been found to inhibit macro and microautophagy^21^, ^22^, ^23^ and control the MVB pathway‐driven degradation of plasma membrane proteins,^24^, ^25^, ^26^, ^27^ through mechanisms that have not been yet characterized.

Multiprotein complexes such as TORC1 in yeast are tethered to the vacuolar membrane and function by integrating signals from many intracellular and extracellular cues from a variety of kinases, GTPases and their effectors.^28^ Recently, an upstream regulator of the TORC1, the yeast SEA complex (GATOR complex in humans), was identified and shown to be part of this web of GTPase effectors.^29^, ^30^ The SEA complex is a conserved eight protein complex (Sea1, Sea2, Sea3, Sea4, Seh1, Sec13, Npr2, Npr3) made up of proteins with structural characteristics similar to the membrane coating complexes such as the nuclear pore complex, the COPII vesicle coating complex and HOPS/CORVET tethering complexes.^31^ The SEA complex is also dynamically associated to the vacuolar membrane; however, its complete function is not well understood.

Substantial effort has gone into understanding the membrane trafficking events required to form autophagosomes and the contributions of SNX‐BAR proteins to the autophagy pathway. In fact, SNX‐BAR proteins have been shown to mediate an emerging number of Atg processes. For example, the Snx4‐Snx41 SNX‐BAR heterodimer mediates the retrograde endosome‐to‐Golgi transport of the Atg protein Atg27, an integral membrane protein that when deleted leads to decreased autophagosome number and autophagic flux in budding yeast.^10^, ^32^ Moreover, Snx4‐mediated retrograde trafficking of proteins and lipids helps the cell maintain the phosphatidylserine (PS) and phosphatidylethanolamine (PE) homeostasis, which is required to allow autophagosome fusion with the vacuole, one of the final stages of autophagy.^11^ As we come to understand the full range of SNX‐BAR protein functions, it is becoming clear that this group of proteins has a critical role in the network of cellular players that collectively regulate autophagy, including those associated with the TORC1 complex.

In this study, we report the identification of a novel yeast SNX‐BAR protein, encoded by yeast open reading frame (ORF) YKR078W, that contributes to TORC1 regulation alongside the SEA complex. Our findings bring us to a more complete understanding of SNX‐BAR family of proteins and the important role they play in TORC1 signaling during autophagy.

## 2 RESULTS

### 2.1 YKR078W/VPS501 and VPS5 are phylogenetically related but functionally distinct

**FIGURE 1:**
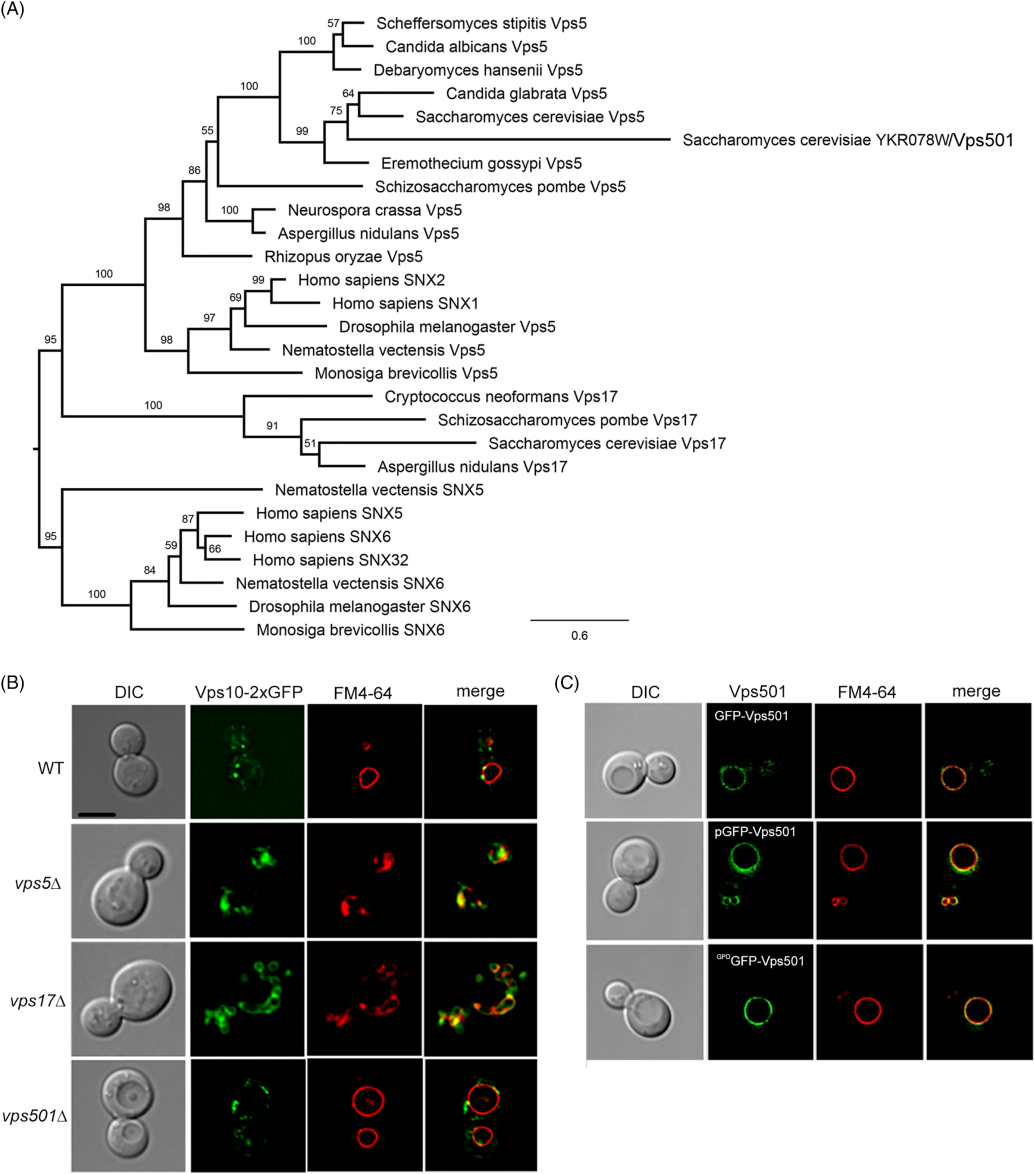
YKR078W/Vps501 is a paralog of Vps5 and resides on the vacuolar membrane. (A) Phylogenetic analysis of Vps5‐like SNX‐BAR proteins in selected animals, choanoflagellate and fungi, including other species in the family Saccharomycetaceae indicates the presence of a Vps5‐like protein (YKR078W) in *Saccharomyces cerevisiae*, referred to here as Vps501. (B) Micrographs of Vps10‐2XGFP in wildtype and indicated mutant cells. Vps501 does not have a role in Vps10 trafficking, despite the phylogenetic similarities with retromer SNX‐BARs. (C) Vps501 localizes to the vacuolar membrane as a N‐terminal GFP fusion protein. GFP‐Vps501 expression is shown as locus integrations using native promoter (top), GPD promoter (bottom) or ectopically expressed as a 2‐micron plasmid (middle). C‐terminal fusions were found to be non‐functional, not shown. Vacuolar membranes are shown using FM4‐64 dye. The scale bar indicates 5 μm. See also Figure S1. BAR, Bin‐Amphiphysin‐Rvs; GFP, green fluorescent protein; GPD, glyceraldehyde‐3‐phosphate dehydrogenase; SNX, sorting nexin

Phylogenetic analysis of the SNX‐BAR SNXs allowed us to identify ORF YKR078W from *S. cerevisiae* as a SNX‐BAR candidate that forms a well‐supported clade (bootstrap = 99) with Vps5 proteins from *S. cerevisiae* and other closely related species from the family Saccharomycetaceae (Figure 1A; Figure S1). As previously shown by Koumandou et al.,^33^ fungal Vps5 proteins are most closely related to SNX1/2 proteins from animals and choanoflagellates.^33^ The simplest explanation for the existence of two Vps5‐like proteins in *S. cerevisiae* is a recent gene duplication event. To assess whether or not there is functional overlap between the Vps5 and YKR078W proteins, we used *YKR078WΔ* yeast cells to examine the localization of the vacuolar hydrolase receptor Vps10. This receptor is trafficked from the prevacuolar endosome to Golgi by the Vps5‐dependent retromer‐SNX‐BAR complex and is mistrafficked to the vacuole in *vps5 Δ* cells.^34^, ^35^, ^36^ We hypothesized that, if there is functional overlap between the Vps5 and YKR078W proteins, Vps10 mislocalization in *YKR078W*Δ would phenocopy the *vps5*Δ mutant. However, deletion of YKR078W did not affect Vps10 localization (Figure 1B), indicating that while Vps5 and YKR078W are phylogenetically related, they are functionally different. In light of these findings, we gave YKR078W the distinct, but Vps5‐related name of Vps501. We will use the Vps501 annotation throughout the remainder of this paper.

### 2.2 N‐terminally tagged Vps501 localizes to the vacuolar membrane

While Vps501 shares common evolutionary ancestors and lineage with Vps5, its function within the cell has so far remained uncharacterized. Multiple genome‐wide localization screens using C‐terminally fused green fluorescent protein (GFP) failed to detect expression and/or failed to localize Vps501 to any intracellular compartment.^37^ Interestingly, we have discovered that Vps501 appears to be nonfunctional as a C‐terminal GFP fusion (data not shown). However, a fully integrated N‐terminal GFP fusion appeared to preserve the functionality and localization of Vps501. Instead of an endosomal localization, which might be expected based on its homology to Vps5, we detect the protein predominantly on the limiting membrane of the vacuole, and in a few intracellular puncta (Figure 1C, upper panels). This localization pattern remains consistent when Vps501 is chromosomally tagged under the control of either its endogenous promoter (Figure 1C, upper panels) or a constitutive TDH3/glyceraldehyde‐3‐phosphate dehydrogenase (GPD) promoter (Figure 1C, lower panels), and when expressed from an extrachromosomal plasmid (Figure 1C, middle, panels).

### 2.3 Identification of Vps501 physical and genetic interactions

**FIGURE 2:**
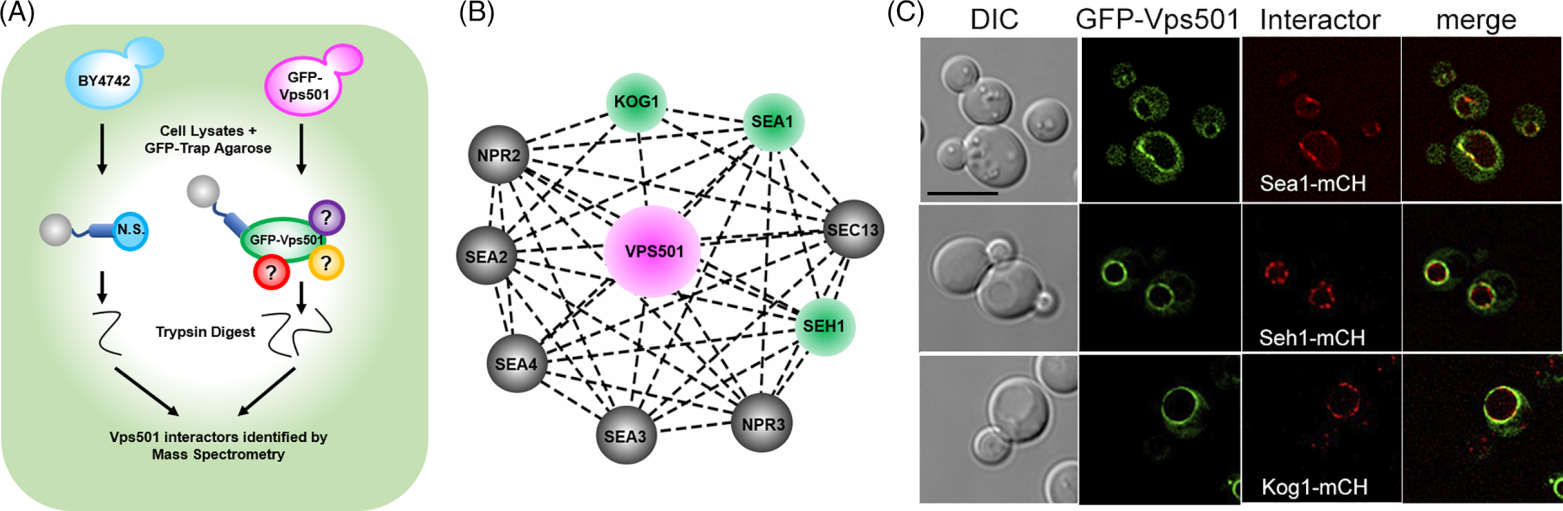
Vps501 interacts with subunits of TORC1 and the SEA complex. (A) Mass spectrometry experimental design to identify Vps501 interactors. GFP‐Vps501 and interactors were purified by GFP‐Trap affinity purification and SDS‐PAGE, followed by in‐gel trypsin digestion. Resulting peptides were analyzed and identified by LC–MS/MS according to their relative enrichment. (B) Mass spectrometry analysis using STRING software identified strong interactions with Kog1, a subunit of the TORC1 complex and Sea1 and Seh1, subunits of the SEA complex. Subunits of the SEA complex and TORC1 are represented as nodes in the graphical network. Proteins colored in green (Sea1, Seh1 and Kog1) are those detected by this proteomic study. Lines connecting the nodes represent previously reported interactions. (C) Micrographs show GFP‐Vps501 colocalizes with Sea1‐mCherry, Kog1‐mCherry, Seh1‐mCherry at the vacuolar membrane. The scale bar indicates 5 μm. TOR, target of rapamycin

To gain insights into Vps501 function, we used a co‐immunoprecipitation mass spectrometry approach to identify its interactors. We purified GFP‐Vps501 complexes and characterized the Vps501 interactome using mass spectrometry (Figure 2A). The list of Vps501 interacting proteins was enriched for proteins involved in amino acid/carbohydrate metabolism, protein translation, protein folding, and endocytosis (Table S1). Our analysis of strong interactors of Vps501 identified subunits of the evolutionarily conserved eight‐protein SEA complex (Sea1, Seh1) and the TORC1 subunit Kog1, each of which resides on the vacuolar membrane and is linked with autophagy through TORC1 signaling regulation (Figure 2B). Moreover, we found that Vps501 and its vacuolar interactors identified through mass spectrometry (Sea1, Seh1 and Kog1), colocalize at the vacuolar membrane (Figure 2C).

**FIGURE 3:**
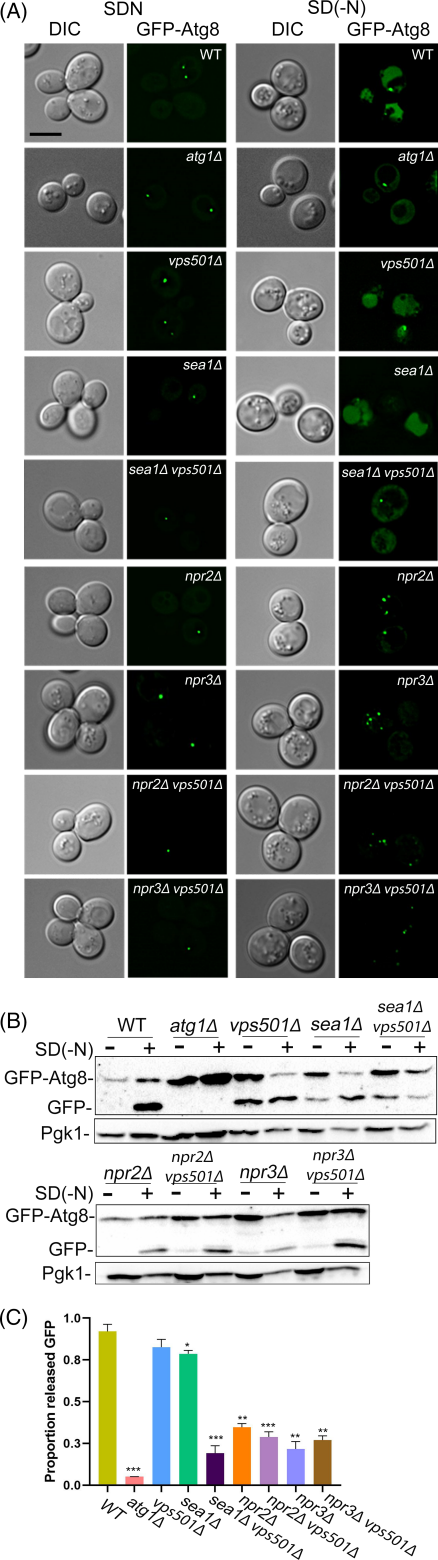
*vps501*Δ*sea1*Δ cells display a synthetic autophagy defect. (A) Maximum projection micrographs of cells expressing GFP‐Atg8 in wildtype and indicated mutant cells before and after nitrogen starvation. GFP‐Atg8 is found in the vacuole lumen (VL) in wildtype cells after nitrogen starvation, indicating successful autophagic flux has occurred. In *atg1*Δ cells, autophagy induction is inhibited and GFP‐Atg8 is absent in the VL. A similar phenotype is also seen only when cells are ablated for both Vps501 and Sea1, indicating a synthetic genetic interaction between Vps501 and Sea1 is critical for autophagy. Cells ablated for Npr2 and Npr3 also show major autophagy defects as single knockouts and likely mask any synthetic phenotypes combined with Vps501 ablation. The scale bar indicates 5 μm. (B) Quantitative immunoblotting was used to detect the amount of GFP‐Atg8 flux before and after autophagy induction. There is a 3‐fold decrease in GFP‐Atg8 flux in *vps501*Δ*sea1*Δ cells, compared to single deletions, whereas there was no significant difference detected in Npr2 or Npr3 knockout cells. A representative immunoblot is shown. Anti‐Pgk1 was used as a loading control. (C) Graph of quantification of GFP‐Atg8 processing. The results are from three experiments and averaged using the standard error of the mean. Indicated significance is a comparison of wildtype to single deletions or double mutants. *p < 0.05, **p < 0.01, ***p < 0.001 indicates significance as calculated by Student's *t*‐test. See also Figure S3

We next performed a series of genetic and functional studies to interrogate the potential link between the *VPS501* gene and TORC1 signaling and to determine whether it is epistatic to any of the genes encoding the subunits of the SEA complex. Nitrogen starvation or rapamycin inhibits TORC1 triggering autophagy induction, and we initially used fluorescence microscopy to assess localization of the canonical autophagy marker GFP‐Atg8 in strains deleted for *VPS501* singly or in combination with different SEA complex subunits upon the induction of autophagy (Figure 3A). GFP‐Atg8 processing was also monitored by immunoblotting to detect increases in both total GFP‐Atg8 signal and ratio of free GFP to total GFP signal over time (Figure 3B,C). Collectively, these assays allowed us to evaluate whether deletion of *VPS501* results in autophagic flux defects or exacerbates those caused by SEA complex mutants. We found that cells lacking *VPS501* display no defects in starvation‐induced or rapamycin‐induced autophagy, unless combined with deletions of SEACIT subunits. Interestingly, both fluorescence microscopy and immunoblot analysis of *sea1*Δ*vps501*Δ cells showed a striking loss of autophagic flux in GFP‐Atg8, indicating that Vps501 and the SEA complex work synergistically during autophagy (Figure 3; Figure S3). Other groups have reported kinetic delays in autophagic flux following deletion of *SEA1*, either alone or in combination with deletion of other SEA complex subunits.^29^ We found that combined loss of *VPS501* and *SEA1* severely impairs autophagy, nearly approximating the complete loss observed in the *atg1Δ* control. It is also worth noting that, as reported in previous studies, *NPR2* and *NPR3* deletions cause significant defects in both bulk and specific forms of autophagy, likely masking any synthetic defect present in *vps501*Δ mutant cells.^38^ *VPS501* deletion in combination with each of the SEACAT subunits resulted in only a partial reduction in autophagic flux as evidenced by GFP‐Atg8 processing defects, with the most significant defect occurring in *seh1Δvps501Δ* cells (Figure S2).

### 2.4 Vps501‐Sea1 interaction stabilizes the SEA complex

**FIGURE 4:**
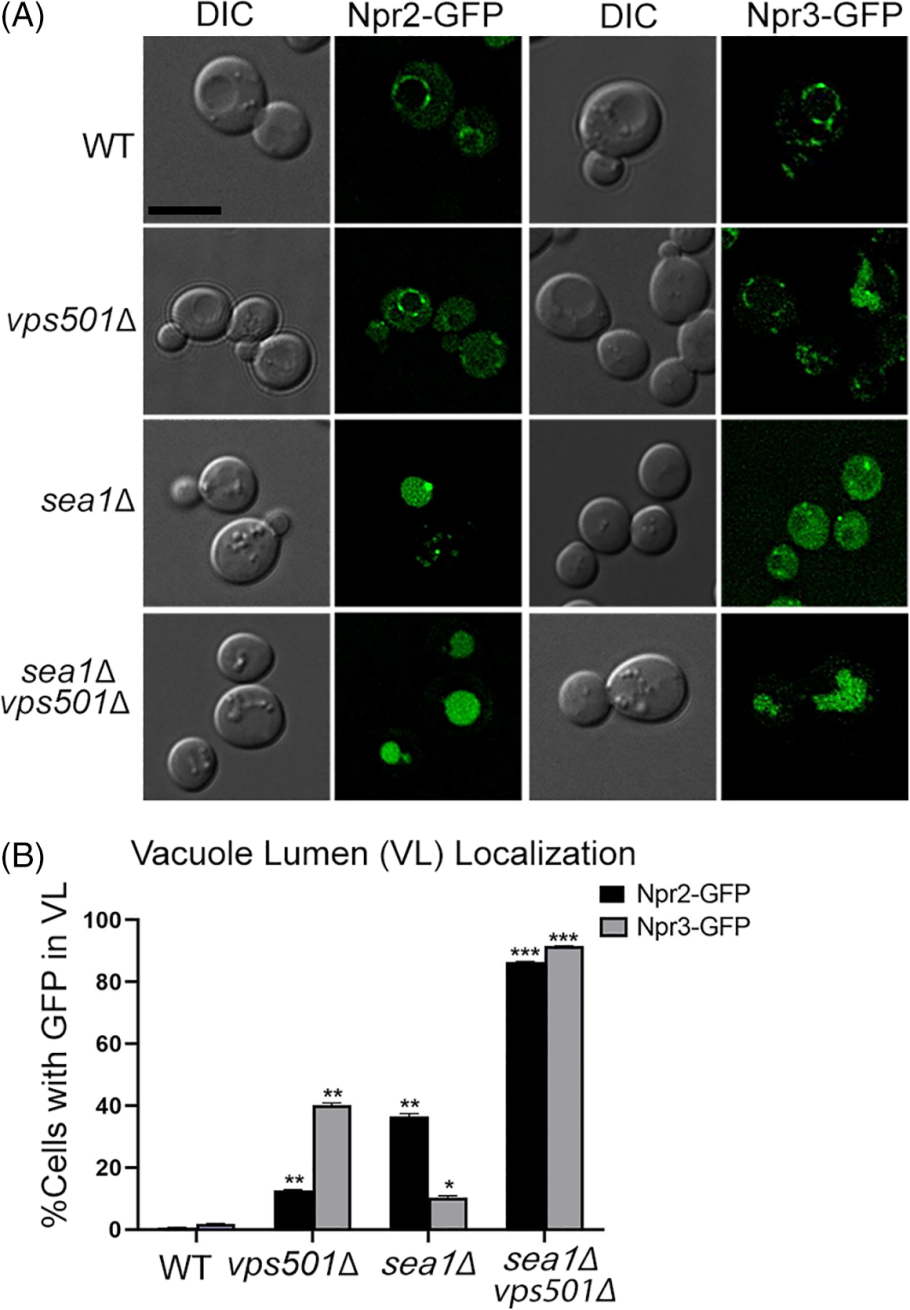
SEACIT subunits, Npr2 and Npr3 require Vps501 and Sea1 for vacuolar localization. (A) Npr2‐GFP and Npr3‐GFP localize to the vacuolar membrane in wildtype cells. In *vps501*Δ or *sea1*Δ cells, Npr2‐GFP and Npr3‐GFP are mislocalized to the vacuole lumen (VL) in 10%–40% of cells. In *vps501*Δ*sea1*Δ cells, Npr2‐GFP and Npr3‐GFP are mislocalized to the VL in 85% of cells. (B) Graph represents VL localization in wildtype cells or mutants, defined by visually scoring the presence of GFP in the vacuole lumen. The results are from three experiments and averaged using the standard error of the mean. Indicated significance is a comparison of wildtype to single deletions or double mutants. *p < 0.05, **p < 0.01, ***p < 0.001 indicates significance as calculated by Student's *t*‐test

Given the strong autophagic defects associated with the combined loss of *Vps501* and the SEA complex, we set out to examine the role of Vps501 in the maintaining the SEA complex to the vacuolar membrane. Again, we found a clear cooperative role for Vps501 and Sea1 to maintain the SEA complex to the vacuolar membrane. Among the SEA complex, we found that the SEACIT subunits Npr2 and Npr3 were dramatically mislocalized to the vacuolar lumen (VL) in 85% of *vps501*Δ*sea1*Δ cells compared to 15%–40% when Sea1 or Vps501 is ablated alone (Figure 4A,B). While the SEACAT subunits Sea2, Sea3, Sea4 were also mislocalized to the VL in 100% of *vps501*Δ*sea1*Δ cells, this defect was masked by the 80%–90% mislocalization observed in the *sea1*Δ single mutant (Figure S4). The remaining SEACAT subunits Seh1 and Sec13 were significantly mislocalized from the vacuolar membrane to non‐vacuolar compartments in both *sea1*Δ and *vps501*Δ*sea1*Δ cells but were not found in the vacuole lumen. Seh1 and Sec13 have previously reported roles in the nucleus and endoplasmic reticulum, respectively, and likely become enriched at these locations when vacuolar membrane localization is compromised (Figure S4).^39^ The enhancement of the phenotype observed in the *vps501*Δ*sea1*Δ cells suggests Sea1 and Vps501 have parallel or partially redundant roles to stabilize the localization of the SEA complex to the vacuolar membrane and in particular the Npr2, Npr3 SEACIT subunits. These results suggested Vps501 is possibly a part of a larger mechanism that regulates TORC1 signaling.

### 2.5 The TORC1 complex and induction of autophagy are defective in vps501Δsea1Δ cells

**FIGURE 5:**
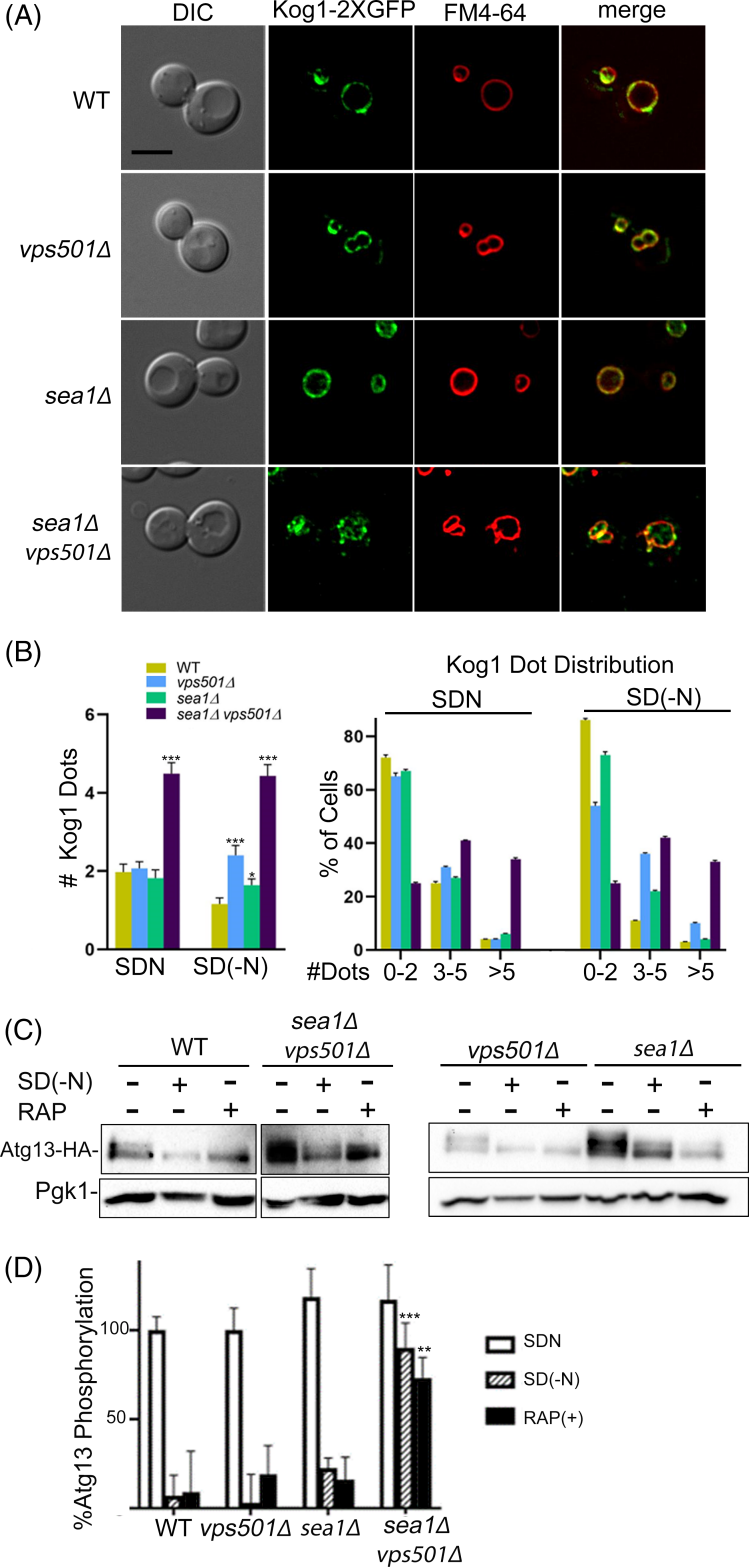
TORC1 and autophagy induction is defective in *vps501*Δ*sea1*Δ cells. (A) Micrographs of Kog1‐2XGFP localization in wildtype and indicated mutant cells during vegetative growth. Kog1 a subunit of TORC1, has previously been reported to localize to the vacuolar membrane and to dot‐like structures juxtaposed the vacuolar membrane. In *vps501*Δ*sea1*Δ cells, similar dot‐like structures accumulate suggesting the non‐vacuolar membrane pool of Kog1 are enriched, while the vacuole membrane TORC1 pool appears reduced. Vacuolar membranes are shown using FM4‐64 dye. (B) The number of Kog1 dot‐like structures were quantified in wildtype and indicated mutant cells before and after nitrogen starvation as described in the text. Regardless of starvation conditions, there was a 2‐fold increase in dot‐like structures in *vps501*Δ*sea1*Δ cells as compared to single mutants or wildtype cells (left graph). Three or more dot‐like structures were found in 80% of *vps501*Δ*sea1*Δ cells, while single mutants or wildtype cells typically had 0–2 dots (right graph). *p < 0.05, **p < 0.01, ***p < 0.001 indicates significance as calculated by a one‐way ANOVA from three biological replicates. (C) Quantitative immunoblot analysis of Atg13 in wildtype and indicated mutant cells before and after nitrogen starvation or rapamycin treatment. In wildtype, *vps501*Δ *or sea1*Δ cells, Atg13 is phosphorylated as indicated by Atg13 band smear during vegetative growth, indicating TORC1 is active. In autophagy induction conditions, TORC1 is inactivated and Atg13 is not phosphorylated indicated by the loss of Atg13 band smear. In *vps501*Δ *sea1*Δ cells, Atg13 remains phosphorylated indicating autophagy induction is defective in these mutants, regardless of nitrogen starvation or rapamycin (see text for details). (D) Percentage of Atg13 phosphorylation was quantified by determining the proportion of Atg13 to total protein using densitometry. A representative immunoblot is shown. Anti‐Pgk1 was used as a loading control. Indicated significance is a comparison of wildtype to single deletions or double mutants. *p < 0.05, **p < 0.01, ***p < 0.001 indicates significance as calculated by Student's *t*‐test from three biological replicates. TOR, target of rapamycin

While other SNXs have previously been found to contribute to autophagy indirectly by mobilizing lipid membranes for autophagosome biogenesis or potentiating vacuolar fusion, the role of Vps501 in autophagy appears to be more direct.^11^, ^40^, ^41^, ^42^ The clear physical and genetic interactions of Vps501 with the SEA complex indicate a potential role in regulating TORC1 signaling, possibly during the induction of autophagy. To test this hypothesis, we targeted subunits of TORC1 to determine whether they were defective in v*ps501*Δ*sea1*Δ cells. We were particularly interested in the Kog1 subunit, as it was one of our most abundant hits in the proteomics screen for Vps501 interactors (Figure 2). Like what others have found, we localized Kog1‐2XGFP to two independent TORC1 pools; one around the vacuolar membrane and a second in dot‐like perivacuolar structures (Figure 5A).^43^, ^44^ This dual localization is consistently present in wildtype, *vps501*Δ *or sea1*Δ cells. Interestingly, the dot‐like perivacuolar structures exhibit enhanced accumulation in *vps501*Δ*sea1*Δ cells, with the average number of Kog1‐2XGFP dots enriched 2‐fold relative to the wildtype or single mutant controls. The vacuolar Kog1‐2XGFP pool appears be reduced, either in the presence or absence of nitrogen (Figure 5A,B). Recently, these dot‐like TORC1 structures have been referred to as signaling endosomes and have been shown to have unique phosphorylation targets.^43^, ^44^

One such target is Atg13, a regulatory subunit of the Atg1 signaling complex that is required for induction of autophagy.^45^ When TORC1 is active, Atg13 is phosphorylated, inhibiting induction of autophagy. Therefore, we hypothesized that Atg13 phosphorylation would be defective in *vps501*Δ*sea1*Δ cells if autophagy induction were impaired. We analyzed Atg13 phosphorylation by immunoblotting in wildtype and mutant cells, both before and after nitrogen starvation or with rapamycin treatment, and quantified the resulting signals. In wildtype, *vps501*Δ *or sea1*Δ cells, Atg13 is clearly phosphorylated as indicated by a smear of the Atg13 band during vegetative growth, demonstrating that TORC1 is active (Figure 5C,D). Likewise, during autophagy‐inducing conditions TORC1 is inactive, and the Atg13 band collapses in wildtype, *vps501*Δ, *or sea1*Δ cells indicating that the majority of Atg13 is not phosphorylated (Figure 5C,D). Interestingly, while nitrogen starvation and rapamycin treatment both trigger TORC1 inactivation, they do so by different mechanisms. Under starvation conditions, low amino acid levels are communicated to TORC1 via the Gtr1/Gtr2 complex, part of a highly conserved family of Rag GTPases, which assembles as heterodimeric complexes on vacuolar membranes and are regulated by their guanine nucleotide loading status to active TORC1 via the SEA complex.^46^ However, rapamycin acts by binding rapamycin binding protein, Fpr1 in yeast and the Fpr1‐rapamycin complex directly binds to TORC1 to inactivate it, thereby bypassing any SEA complex inhibition.^47^, ^48^ Surprisingly, Atg13 remained phosphorylated in *vps501*Δ*sea1*Δ cells, regardless of nitrogen starvation or rapamycin treatment, indicating autophagy induction is defective in these mutants, (Figure 5C,D). Moreover, this suggests the vacuolar pool of TORC1 is not accessible to the Fpr1‐rapamycin inhibition in *vps501*Δ*sea1*Δ cells, likely resulting in a broad TORC1 signaling defect independent of autophagy induction.

To test this hypothesis, we visualized two additional vacuolar membrane proteins to determine if their distribution is also affected in *vps501*Δ *or sea1*Δ cells. Atg27 is an Atg transmembrane protein and was chosen for investigation as a potential mechanistic link because of its characterized vacuolar‐endosomal trafficking itinerary.^49^ In wild‐type cells, Atg27‐2XGFP cycles between the vacuolar membrane, Golgi/endosome and autophagic compartments. In *vps501∆sea1∆* cells, we found that Atg27‐2XGFP is significantly depleted from the vacuolar membrane, indicating TORC1 signaling maybe responsible for maintaining Atg27 at the vacuolar membrane (Figure S5). To assess non‐autophagy related TORC1 pathway integrity, we also imaged Ypt7 in the mutant backgrounds of interest. Ypt7 is a Rab family GTPase that is required for endosome‐endosome fusion and localizes the vacuolar membrane.^50^ Ypt7‐mCherry was also notably depleted from the vacuolar membrane when Vps501 and Sea1 are ablated singly or together, suggesting aberrant TORC1 hyperactivity is also affecting Ypt7‐dependent cellular pathways (Figure S5). However, it is not yet clear whether this mechanism is mediated by a direct or indirect interaction with Vps501 and the SEA complex or is an overall consequence of a broad TORC1 misregulation.

### 2.6 Vps501 requires Sea1 and a non‐canonical PX domain for vacuolar membrane localization and function

**FIGURE 6:**
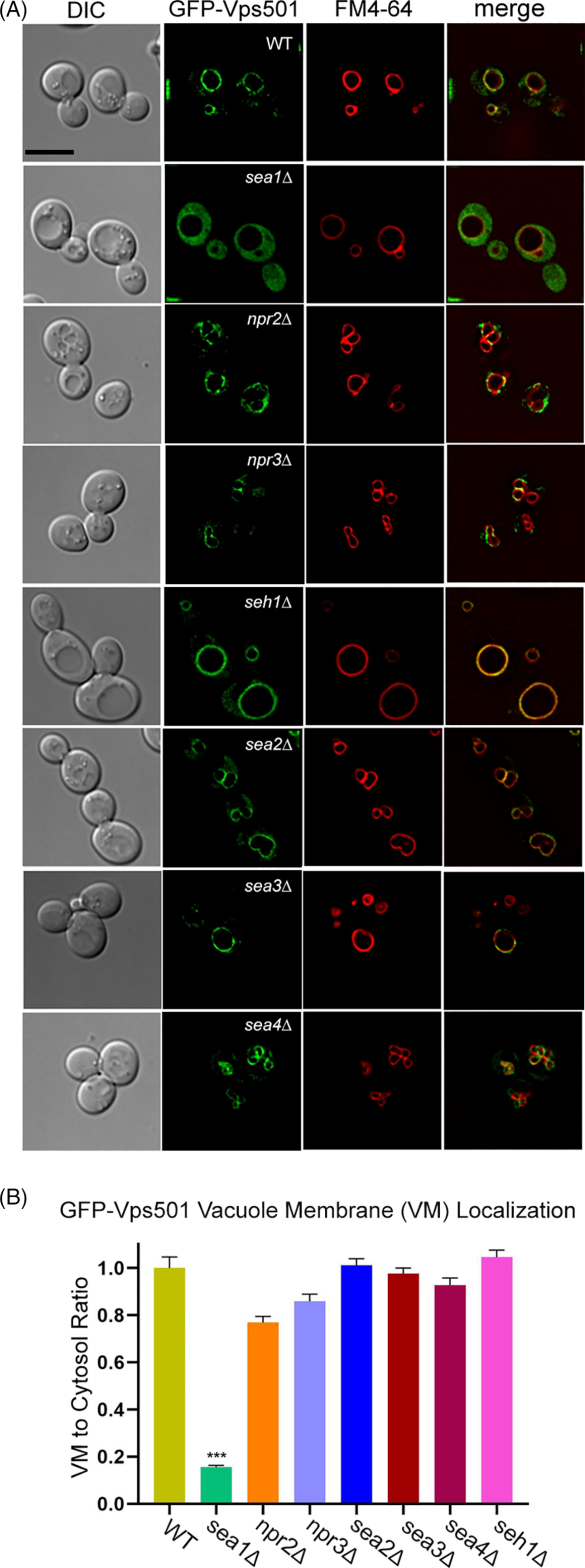
Vps501 requires Sea1 for vacuolar localization. (A) Micrographs of GFP‐Vps501 in wildtype and SEA complex mutant cells. GFP‐Vps501 is significantly mislocalized in SEACIT mutant cells but not in SEACAT mutant cells. *sec13*Δ cells are nonviable. (B) Vacuolar membrane localization was quantified by comparing GFP‐Vps501 VML/cytosol ratios in wildtype versus SEA complex mutants. A minimum of 100 cells were measured in triplicate; standard error of the mean was calculated. *p < 0.05, **p < 0.01, ***p < 0.001 indicates significance as calculated by Student's *t*‐test

**FIGURE 7:**
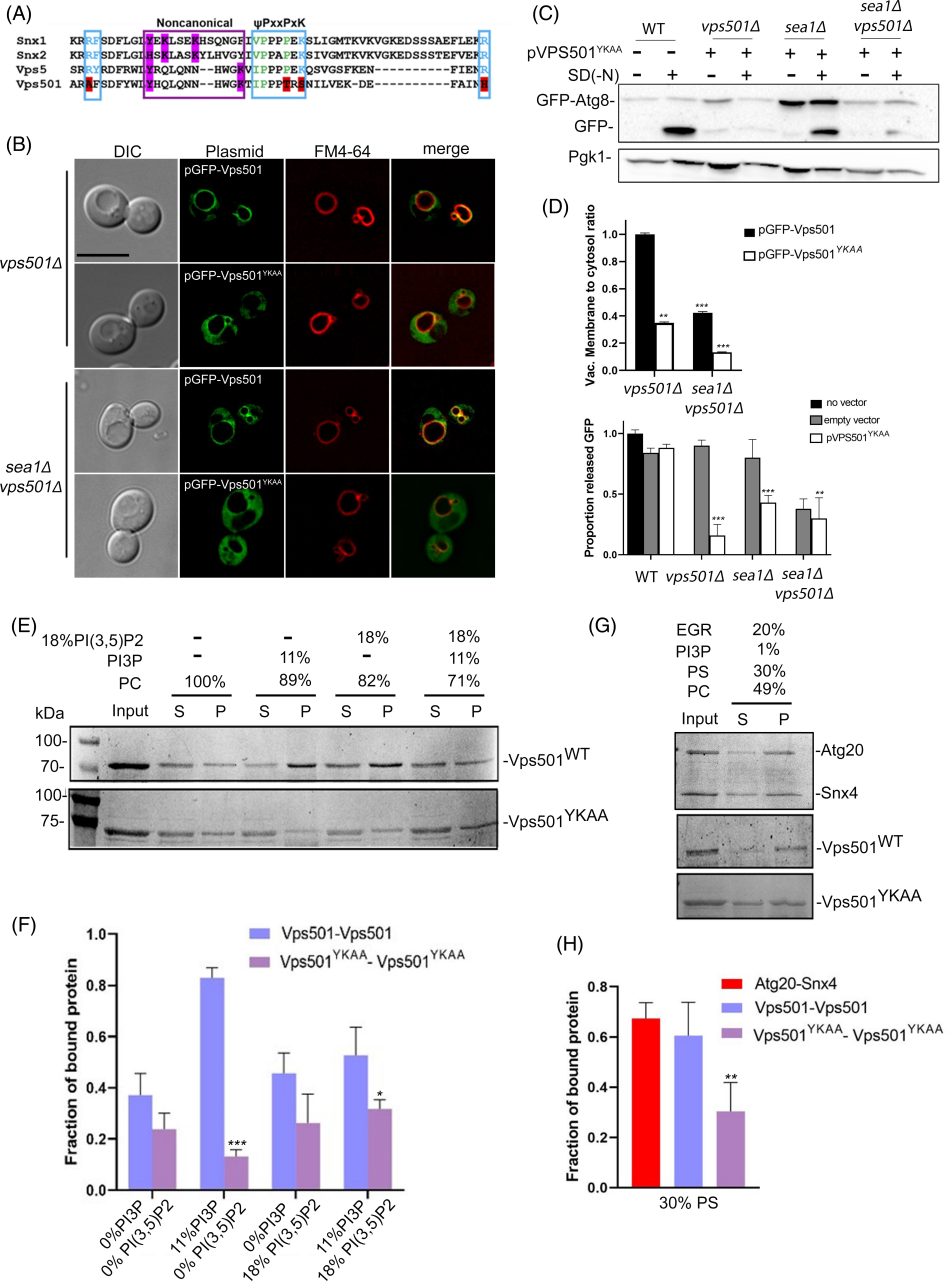
Vps501 non‐canonical Phox (PX) domain is required for localization and function at the vacuolar membrane. (A) Sequence alignments of human SNX1, SNX2 and yeast homologs VPS5 and VPS501 PX domains are shown. Blue indicates residues in the canonical PI3P motif. Magenta indicates key secondary lipid binding site residues. Vps501 canonical PI3P motif is not conserved, however, the non‐canonical His/Tyr motif is conserved. (B) Alanine mutations to His/Tyr residues in the non‐canonical motif resulted in GFP‐Vps501^YKAA^ mislocalized to the cytosol in *vps501∆*, and *vps501∆sea1∆* cells, indicating Vps501's recruitment to the vacuolar membrane is dependent on both PI3P recognition and Sea1 binding. The scale bar indicates 5 μm. (C) Ectopic expression of Vps501^YKAA^ failed to complement *vps501∆sea1∆* cells in GFP‐Atg8 processing assays and severely impairs autophagy flux when expressed in *sea1∆* cells. (D) Top Graph, quantification of Vps501 localization was defined by vacuolar membrane to cytosol ratios as described in material and methods. GFP‐Vps501^YKAA^ recruitment to the vacuolar membrane is reduced by ~60% in *vps501Δ* cells. When combined with ablation of Sea1 vacuolar membrane localization is reduced by ~90%. Bottom Graph, quantification of GFP‐Atg8 processing when Vps501^YKAA^ is ectopically expressed in wildtype, *vps501∆*, or *vps501∆sea1∆* cells. The results are from three experiments and averaged (standard deviation). (E and F) PI liposome sedimentation assays and quantification. Query proteins and lipids are indicated on the figure. Vps501^WT^ bound to liposomes enriched with PI3P or PI(3,5)P. Binding was reduced when PI3P and PI(3,5)P lipids were combined on liposomes. (G and H) PS liposome sedimentation assays and quantification. Yeast SNX‐BARs heterodimer Snx4‐Atg20 has previously been shown to preferentially bind PS‐containing membranes. Vps501^WT^ also strongly binds to liposomes containing 30% PS, similar to Snx4‐Atg20, whereas Vps501^YKAA^ PX mutant binding was reduced 2‐fold. Indicated significance is a comparison of wildtype to single deletions or double mutants. *p < 0.05, **p < 0.01, ***p < 0.001 indicates significance as calculated by Student's *t*‐test. SNX, sorting nexin

Due to Vps501's unique vacuolar localization and apparent interactions with vacuolar proteins, we hypothesized that direct recruitment via the SEA complex is critical for Vps501 vacuolar localization. Indeed, we found that ablation of Sea1 causes mislocalization of GFP‐Vps501 to the cytoplasm (Figure 6). Interestingly, deletion of any other SEA complex subunit failed to trigger mislocalization of GFP‐Vps501 from the vacuole, suggesting that an interaction with Sea1 specifically mediates Vps501 recruitment to the vacuolar membrane. We next aimed to determine whether the vacuolar membrane localization of Vps501 is required for its autophagic function. In order to generate a mutant Vps501 protein that is unable to localize to the vacuolar membrane, we sought to first identify the domain responsible for this localization. We began by examining the lipid‐binding PX domain that might mediate this localization through recognition of the specific lipid composition. Although the canonical lipid‐binding motif in the PX domain of Vps501 is missing key motif residues that would be required for PI3P lipid binding, we discovered a predicted secondary noncanonical binding motif (Figure 7A). We used site‐directed mutagenesis of the pGFP‐Vps501 plasmid to disrupt the noncanonical lipid binding motif to determine its effect on localization of Vps501 to the vacuole. We substituted alanine for the key conserved tyrosine and lysine residues in the noncanonical lipid binding motif of Vps501, generating a mutant protein referred to here as GFP‐Vps501^YKAA^. Colocalization studies of pGFP‐Vps501^YKAA^ and the vacuolar membrane marker FM4‐64, indicated that GFP‐Vps501^YKAA^ recruitment to the vacuolar membrane is reduced by 70% relative to the GFP‐Vps501 control (Figure 7B,D, upper graph). This 70% reduction in the vacuolar membrane recruitment of pGFP‐Vps501^YKAA^ was seen in both *vps501∆* and *sea1∆* cells, and combination of pGFP‐Vps501^YKAA^ with *vps501∆sea1∆* led to a near total loss of Vps501 vacuolar localization (Figure 7B,D, upper graph). This indicated that both protein–protein interaction with Sea1 and lipid recognition by the lipid‐binding motif are important for Vps501 localization to the vacuolar membrane (Figure 7B,D, upper graph). We next ectopically expressed *VPS501* ^*YKAA*^ from a 2‐micron plasmid and found that the mutant fails to complement *vps501∆sea1∆* cells in GFP‐Atg8 processing assays (Figure 7C). Taken together with the severe defect in *sea1∆* cell autophagic flux associated with the *VPS501* ^*YKAA*^ mutant relative to the wild‐type control (Figure 7D, upper graph), the combination of Sea1 interaction and lipid‐binding appears to be required not only for Vps501 recruitment to the vacuolar membrane but also for its role in autophagy and TORC1 signaling.

### 2.7 Vps501 lipid binding specificity

Next, we tested whether Vps501 preferentially binds PI3P or PI(3,5)P2, the prevalent phosphoinositides (PI) on the vacuolar membrane. To do this we performed liposomes sedimentation studies using liposomes empirically derived from vacuole lipid composition or control liposomes devoid of PI3P or PI(3,5)P2.^51^, ^52^ We tested recombinantly expressed Vps501^WT^ and the Vps501^YKAA^ PX mutant, and found strong binding affinities to liposomes enriched with PI3P when using Vps501^WT^ but a 9‐fold reduction in binding for the Vps501^YKAA^ PX mutant (Figure 7E,F). We also observed some minor binding enhancements when PI(3,5)P2 was present alone or in combination with PI3P as compared to control liposomes (Figure 7E,F). However, Vps501^WT^ binding was 2‐fold lower as compared to liposomes with only 11% PI3P, suggesting PI(3,5)P2 may have an inhibitory effect on Vps501 binding. Recently, we discovered other yeast SNX‐BARs Snx4 and Atg20 heterodimerize and preferentially bind PS‐containing membranes; however, this was not the case for the Vps5‐Vps17 retromer SNX‐BARs, or for Mvp1, a homodimeric SNX‐BAR that functions on the retromer pathway.^11^ Given the reported presence of PS on the vacuolar membrane we sought to determine if Vps501 possessed similar PS binding preferences.^52^ Indeed, we found Vps501^WT^ strongly binds to liposomes containing 30% PS, similar to Snx4‐Atg20, whereas Vps501^YKAA^ PX mutant binding was reduced 2‐fold (Figure 7G,H). Taken together, we conclude Vps501 vacuolar membrane binding is likely driven by strong binding preferences to PI3P and PS, but overall vacuole recruitment maybe regulated or transiently affected by PI(3,5)P2.

## 3 DISCUSSION

In this study, we report the identification of a novel vacuolar membrane SNX‐BAR protein, Vps501. While phylogenetically, Vps501 is most related to Vps5, an essential component of the yeast retromer complex, protein sequence analysis shows residue variations acquired during its divergence (Figure 1; Figure S1). In this study, we demonstrate how divergence from Vps501 and Vps5 has resulted in unique functional differences. For example, current models indicate that an evolutionarily conserved Phox‐homology (PX) domain, a feature common in all SNXs, binds specifically to PI3P, the major phospholipid of the endosome. However, Vps501 has no canonical PI3P binding motif, as it is missing key motif residues. Instead, Vps501 has a secondary site that is solely responsible for lipid binding. Also, Vps501 exclusively resides on the vacuolar membrane, a unique feature among the SNX‐BAR protein family. We speculate Vps501 likely possesses lipid specificity beyond PI3P as was discovered in other SNXs.^4^ This notion was supported by the GFP‐Vps501^YKAA^ mutant mislocalizing to the cytosol, leaving only a small percentage remaining on the vacuolar membrane, like GFP‐Vps501^WT^ in *sea1Δ* cells. Furthermore, Sea1 was found to be critical for Vps501 localization, indicating a physical recruitment to the vacuolar membrane (Figure 7). Interestingly, in *sea1Δ vps501* ^*YKAA*^ cells, the cytoplasmic mislocalization of the Vps501^YKAA^ mutant was completely blocked from the vacuolar membrane, suggesting Vps501 localization requires both the vacuolar membrane lipids and resident vacuolar proteins. Whether these two binding requirements are universal or specialized to each SNX‐BAR protein is not known. However other SNX‐BAR proteins such as Mvp1, an endosomal SNX‐BAR, was recently found to tetramerize and dissociate in order to bind membrane, indicating a selective combination of protein–protein interaction regulation and lipid specificity likely determine the function of each SNX‐BAR.^53^

In this study, we hypothesized the unique lipid profile of the vacuole mediates Vps501 lipid binding. Unlike many other cellular compartments, the vacuole has very low levels of ergosterol and sphingolipids and the major phosphoinositide species on the vacuolar membrane is phosphatidylinositol 3,5‐bisphosphate (PI(3,5)P2), which is generated from PI3P by the Fab1 kinase.^54^ PI(3,5)P2 is critical for maintaining vacuolar identity since PI(3,5)P2 effectors are required for protein sorting and retrograde trafficking from the vacuole to the endosome.^54^ However, in our sedimentation assays, we found Vps501 possesses more lipid specificity to PI3P and PS, while the presence of PI(3,5)P2 appeared to decrease overall affinity, suggesting a possible mechanism of regulation. Future studies will help us understand the novel ways in which Vps501 recognizes vacuolar lipids and how this recognition influences its function and regulation.

Multiple lines of evidence support a role for Vps501 in TORC1 signaling during autophagy induction. First, using a co‐immunoprecipitation mass spectrometry approach, we identified subunits of the evolutionary conserved SEA complex (Sea1, Seh1) and TORC1 subunit Kog1 (Figure 2). Each of these identified proteins resides on the vacuolar membrane and colocalizes with Vps501. It is also worth noting that the previous studies in which mass spectrometry‐based proteomics were used to discover the eight components of the SEA complex all utilized an individual band excision‐based proteomic approach focusing on the most enriched bands.^38^ This design excluded low‐abundant or low‐affinity interactions and may account for why Vps501 was not detected in their screens.

Second, we found that Vps501 works together with the SEA complex to mediate GFP‐Atg8 autophagic flux. We found that cells lacking Vps501 display a severe deficiency in autophagy only when SEA complex subunits were deleted in combination (Figure 3; Figure S2). A recent study came to similar conclusions, showing that loss of Sea2, Sea3 or Sea4 did not trigger major defects unless combined with *sea1Δ*.^29^, ^38^, ^39^ This led us to believe that Vps501 and the SEA complex cooperate within a synergistic pathway during autophagy induction. Furthermore, the complete impairment of autophagic flux in *vps501* ^*YKAA*^ cells suggests that a negative mutation within the vacuolar membrane recognition site of Vps501 is sufficient to drive impairment of the SEA complex (Figure 7).

Last, we have defined the role of Vps501 as a co‐regulator of autophagy that promotes SEACIT inhibition of TORC1 during autophagy induction. We found the SEACIT subunits Npr2 and Npr3 are severely destabilized in *vps501*Δ*sea1*Δ cells. Npr2, Npr3 and Sea1 act as a GTPase‐activating complex to Gtr1 and Gtr2 which in turn mediates TORC1 activation at the vacuolar membrane.^46^ Previous studies have demonstrated that deletion of Sea1 alone resulted in partial mislocalization of Npr2 and Npr3, which causes hyperactivation of TORC1.^30^, ^55^ In our experiments, Npr2 and Npr3 are severely (>90%) destabilized in *vps501*Δ*sea1*Δ cells, more so than any single deletion alone (Figure 4). Therefore, Gtr1 and Gtr2 are unable to regulate their guanine nucleotide loading and TORC1 remains hyperactive in *vps501*Δ*sea1*Δ cells. Recently, it has also been documented that TORC1 initiates microautophagy which downregulates vacuole surface proteins.^21^, ^22^, ^23^ We investigated the possibility of this phenomenon by looking at two other vacuolar membrane proteins Atg27 and Ypt7 and indeed found both are destabilized from the vacuolar membrane in *vps501*Δ*sea1*Δ cells (Figure S5). This result suggested a TORC1 signaling defect beyond autophagy may also be present, which led us to hypothesize a broad TORC1 hyperactivation may be causing these phenotypes.

The yeast Rag GTPase‐TORC1 complex is found in two spatially and functionally distinct pools, on both the vacuolar and endosomal membranes.^43^, ^44^ Vacuolar TORC1 promotes protein synthesis through its proximal effector Sch9, while endosomal TORC1 controls autophagy induction through phosphorylation of Atg13, preventing Atg1 complex formation at the pre‐autophagosomal structure. Interestingly, *vps501*Δ*sea1*Δ cells show depletion of the Kog1‐2XGFP subunit of TORC1 from the vacuolar membrane and enrichment in dot‐like structures by nearly 2‐fold (Figure 7). Likewise, the endosome‐specific TORC1 substrate Atg13 is hyperphosphorylated in *vps501*Δ *sea1*Δ cells (Figure 7). In addition, the autophagy defects observed in *vps501*Δ *sea1*Δ cells could not be bypassed by rapamycin treatment (Figure 5; Figure S3). This was surprising, given rapamycin is a potent lipophilic macrolide antifungal drug that blocks TORC1 signaling via a direct TOR1 inhibition that is independent of SEA complex regulation.^56^, ^57^ This result, combined with Kog1 mislocalization (Figure 5A,B), suggests an unregulated pool of TORC1 is causing broad TORC1 signaling dysfunction, likely an underlying defect in *vps501*Δ*sea1*Δ cells.

**FIGURE 8:**
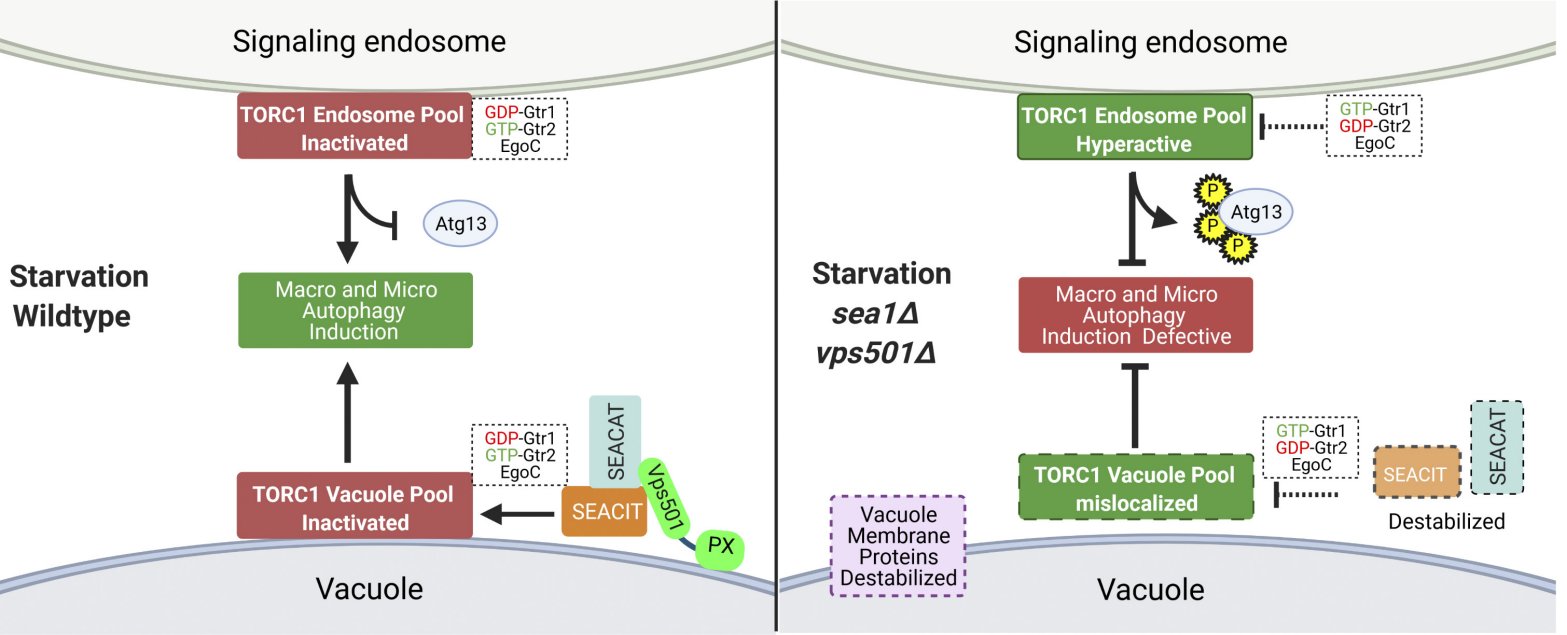
Vps501 cooperates with the SEA complex to inactivate TORC1. Under starvation conditions, Vps501 acts as a stabilizer for the SEA complex to the vacuolar membrane using a non‐canonical PX domain and a direct interaction within the SEA Complex. This interaction promotes SEACIT inactivation of the vacuole and endosomal pools TORC1 via the GTR1/GTR2/EGO complex, resulting in the induction macro and microautophagy. In *vps501*Δ *sea1*Δ cells, the SEA complex is destabilized and the vacuole pool of TORC1 is mislocalized. TORC1 endosomal pools are enriched and hyperactive, resulting in aberrant Atg13 phosphorylation and defective macro and microautophagy which can lead to the overall destabilization of other vacuolar membrane proteins. TOR, target of rapamycin [Correction added on 22 February 2022, after first online publication: Figure 8 has been corrected.]

Collectively, these findings lead us to propose a new model for Vps501 function at the vacuolar membrane (Figure 8). When wildtype cells are deprived of nitrogen, the SEA complex acts through effector molecules such as the Gtr1‐Gtr2/EGO complex to inhibit TORC1 and facilitate autophagic induction. We believe Vps501 acts as a structural stabilizer and tethers the SEA complex to the vacuolar membrane, using multiple interactions within the SEA complex (Sea1 and others) along with lipid specificity from its non‐canonical PX domain to facilitate TORC1 inactivation, thereby inducing autophagy and promoting maintenance of vacuole proteins such as Atg27 and Ypt7. In contrast, *vps501*Δ *sea1*Δ cells exhibit destabilization of the SEA complex, resulting in the loss of the vacuolar pool of TORC1 and a concomitant increase to a non‐vacuolar membrane pool of TORC1 such as signaling endosomes, resulting in TORC1 signaling hyperactivity. This results in broad TORC1 dysfunction in processes such as micro‐ or macroautophagy.

## 4 MATERIALS AND METHODS

### 4.1 Phylogenetic analysis

To determine the identity of *S. cerevisiae* YKR078W in relation to other SNX‐BAR SNXs, we conducted a phylogenetic analysis. We used a former analysis of SNX‐BAR proteins in opisthokonts (e.g., fungi Vps5, and Vps17, animal and choanoflagellate SNX 5/6/32 and SNX 1/2) by Koumandou et al.^33^ as an initial seed alignment. We added additional Vps5 proteins from other fungal species from the family Saccharomycetaceae for better resolution of fungal Vps5 proteins, including YKR078W. Furthermore, to identify if other fungi have a protein similar to YKR078W, we used BLASTp to query protein models at FungalDB to identify additional SNX‐BAR SNXs with similarity to YKR078W from *S. cerevisiae*. These searches only recovered the single Vps5 protein from the fungal species. No additional proteins were detected with an E‐value less than 10 (search threshold). Full‐length sequences for all taxa for each gene family were aligned with Muscle 3.6^58^ and edited manually in the case of clear errors. Maximum likelihood analyses were conducted with RAxML v.8.2.4^59^ using a LG + G matrix model determined by ProtTest v.3,^60^ and a trimmed alignment containing the conserved PX + BAR and SNX‐BAR domains. Support for particular nodes for maximum likelihood analyses was assessed with 1000 bootstraps. Trees were visualized and illustrated with FigTree v1.4 (http://tree.bio.ed.ac.uk/software/figtree/).

### 4.2 Yeast strains and culture conditions

Yeast strains were grown using standard media and conditions unless indicated.^61^ Yeast strains were constructed in BY4742 (*MAT*α *his3‐1*, *leu2‐0*, *met15‐0* and *ura3‐0)* by homologous recombination of gene‐targeted, polymerase chain reaction (PCR)‐generated DNAs using the method of^62^ and/or derived from the EUROSCARF KANMX deletion collection (Open Biosystems/Thermo Scientific) or produced by replacement of the complete reading frame with the *HIS3MX6* or *URA3* cassette. Gene deletions were confirmed by PCR amplification of the deleted locus. Cells were grown in standard synthetic complete medium lacking nutrients required to maintain selection for auxotrophic markers and/or plasmid, unless indicated otherwise. To induce bulk or non‐selective autophagy, cells were grown to log phase, harvested, and transferred to SD(‐N) medium for nitrogen starvation (2% dextrose, 0.17% Yeast Nitrogen Base without amino acids and without ammonium sulfate) for 16 h or resuspended in standard synthetic complete medium containing 0.2 μg/mL of rapamycin (R‐5000, LC laboratories) for 2–4 h at 30°C.

For the construction of an integrated N terminal GFP‐Vps501 yeast strain under a GPD promoter, we PCR amplified pGFP‐^GPD^Vps501 (described below) using the following primers that included 50 bp flanking the *VPS501* locus; ATCAGAACTGCAACCCTACAGATTAGATATGGAGAACGACAAGGCGTCACGTGAGCAAGGGCGAGGAGCTGTTCA and GCTTTTTCAGTAGTAAATTATCTTCTTTAATTACGTTATTATGTACATATTTGGCTTATGTGCTCATCTGGTACA. PCR products were subsequently transformed into cells ablated for *VPS501* (by replacement with a URA marker) and allowed to homologously recombine into the *VPS501* locus. Resulting clones were selected on F.O.A 5‐Fluoroorotic Acid (5‐FOA) and confirmed by PCR.

### 4.3 Light microscopy and image analysis

Yeast cells from cultures grown to OD_600_ ≈ 0.5 were mounted in growth medium, and 3D image stacks were collected at 0.3‐μm z increments on a DeltaVision elite workstation (Cytiva) based on an inverted microscope (IX‐70; Olympus) using a 100 × 1.4NA oil immersion lens. Images were captured at 24°C with a 12‐bit charge‐coupled device camera (CoolSnap HQ; Photometrics) and deconvolved using the iterative‐constrained algorithm and the measured point spread function. To visualize vacuole morphology, yeast cells were labeled with 7‐Aminochloromethylcoumarin (CMAC; Life Technologies) at a concentration of 100 μM for 30 min in synthetic medium at room temperature (RT). To visualize the vacuolar membrane, FM4‐64 (32 nM) was added to cell cultures for 20 min at 30°C. Cells were then washed, resuspended in fresh medium, and then incubated for 60 min to allow FM4‐64 to accumulate in the vacuolar membrane.^63^ Image analysis and preparation was done using Softworx 6.5 (Cytiva) and ImageJ v1.50d (Rasband).

Vacuolar membrane localization analyses for GFP‐Vps501, GFP‐Vps501^YKAA^, Atg27‐2XGFP, YPT7‐mCherry was determined using a manual method implemented using ImageJ v1.53c (Rasband). A region of interest (ROI) was selected to contain a single cell and the total sum of GFP fluorescence was calculated (TF). Next, we used the Mask macro to delineate the vacuole ROI defined by FM4‐64 and overlayed onto the GFP channel to define the vacuole fluorescence (VF). To calculate cytosol fluorescence intensity (CF), the vacuole mask was inverted so that all pixels outside of the mask were assigned a maximum value and the regions corresponding the vacuole signal were assigned a value of zero. A ratio of the VF to TF is presented in the graphs. Vacuolar membrane localization for Seh1‐GFP, Sec13‐GFP and Atg27‐2xGFP, were calculated by calculating the percent of cells with GFP signal on the vacuole using FM4‐64 and CMAC as visual guides to determine vacuole boundaries. To quantify VL localization, wildtype cells or mutants were visually scored for presence of GFP in the VL. Kog1‐GFP puncta numbers were quantified from z stacks collected at 0.3‐μm intervals. Total patches/cell were counted from maximum intensity projections from small budded cells. A minimum of 100 cells was used in all experimental conditions and performed in biological triplicate.

### 4.4 GFP‐Atg8 processing and immunoblotting

For quantitative immunoblot analysis of GFP‐Atg8, cells were grown under standard vegetative or autophagy inducing conditions to OD_600_ ≈ 0.5, as described above. Typically, 3.0 × 10^7^ cells were harvested by centrifugation and lysed by glass bead agitation in SDS‐PAGE sample buffer. 10% polyacrylamide gels were loaded with 5.0 × 10^7^ cell equivalents and transferred onto standard 0.45 μm nitrocellulose. Anti‐GFP primary mouse monoclonal antibody (1814460, Roche) was diluted 1:2500 and Santa Cruz (sc‐2055) goat anti‐mouse HRP‐conjugated antibody was used at 1:10000. Anti‐Pgk1 at 1:5000 (Life Technologies) was used as loading controls. Centromeric GFP‐Atg8^64^ plasmids were used in the processing assays.

Atg13 immunoblots were done as previously described^65^ with the following modifications. 3.0 × 10^7^ cells were harvested by centrifugation and precipitated by Trichloroacetic acid (TCA). 7.5% polyacrylamide gels were loaded with 0.75 × 10^7^ cell equivalents. Anti‐HA monoclonal (Invitrogen, 26 183) and used at 1:5000 and goat anti‐mouse HRP‐conjugated antibody was used at 1:5000. p3xHA‐Atg13 was purchased from Addgene (Plasmid #59544) and used in all indicated experiments. All enhanced chemiluminescence blots were development on a Chemidoc‐MP (Bio‐Rad) and band intensities were quantified using Quantity One 1D analysis software (Bio‐Rad) and all statistical analysis done using GraphPad Prism 8.

### 4.5 Plasmids

pGFP‐^GPD^Vps501 was constructed using Gateway cloning. Plasmid insert was made using PCR amplified wild‐type genomic *VPS501* locus with the following primers: GGGGACAAGTTTGTACAAAAAAGCAGGCTTAGAGAACGACAAGGCGTCACAT and GGGGACCACTTTGTACAAGAAAGCTGGGTTTCATTGGCTTATGTGCTCATCTGGT and cloned using a BP recombination reaction into pDONR221. Resulting DONOR vector was recombined with pAG425GPD‐EGFP‐ccDB^66^ in a final LR recombination reaction to generate the pAG415GPD‐eGFP‐Vps501 expression clone (pGFP‐^GPD^Vps501).

pGFP‐Vps501^YKAA^ was made commercially using site‐directed mutagenesis (GenScript), introducing alanine mutations into following positions Y160A and K170A of pGFP‐^GPD^Vps501. pVps501^YKAA^ was derived from pGFP‐Vps501^YKAA^ using Gateway cloning as described above.

### 4.6 Mass spectrometry

Yeast strains expressing GFP‐Vps501 were grown to 0.5 × 10^7^ cell density in YPD media. GFP fusion proteins were purified as follows: 200 mg of whole cell protein extract was incubated with GFP‐Trap Magnetic Agarose (ChromoTek) at 4°C for 20 min with gentle agitation. GFP‐Trap beads were collected and washed five times (50 mM Tris‐HCL pH 7.4, 150 mM NaCl, Roche complete Protease Inhibitor Cocktail EDTA free). After the final wash, the buffer was aspirated and the GFP‐Trap beads were incubated with 5× SDS‐PAGE sample buffer and denatured for 5 min at 95°C. 20 μL of each sample was analyzed by SDS‐PAGE, followed by an immunoblot. The experiment was performed in triplicate and normalized to the Nano‐Trap magnetic beads alone.

### 4.7 Trypsin digestion of samples from SDS‐PAGE gel plugs

The gel plugs for each sample were excised by a sterile razor blade, divided into two sections 1 cm each, and additionally chopped into 1 mm pieces. Each section was washed in distilled H_2_O and destained using 100 mM NH_4_HCO_3_ pH 7.5 in 50% acetonitrile. A reduction step was performed by addition of 100 mL 50 mM NH_4_HCO_3_ pH 7.5 and 10 mL of 200mMtris(2‐carboxyethyl) phosphine HCl at 37°C for 30 min. The proteins were alkylated by addition of 100 mL of 50 mM iodoacetamide prepared fresh in 50 mM NH_4_HCO_3_ pH 7.5 buffer and allowed to react in the dark at 20°C for 30 min. Gel sections were washed in water, then acetonitrile, and vacuum dried. Trypsin digestion was carried out overnight at 37°C with 1:50–1:100 enzyme‐protein ratio of sequencing grade‐modified trypsin (Promega) in 50 mM NH_4_HCO_3_ pH 7.5, and 20 mM CaCl_2_. Peptides were extracted with 5% formic acid and vacuum dried and sent to the Mayo Clinic Proteomics Core facility for HPLC and LC‐MS/MS data acquisition.

### 4.8 HPLC for mass spectrometry

All samples were re‐suspended in Burdick & Jackson HPLC‐grade water containing 0.2% formic acid (Fluka), 0.1% TFA (Pierce), and 0.002% Zwittergent 3–16 (Calbiochem), a sulfobetaine detergent that contributes the following distinct peaks at the end of chromatograms: MH^+^ at 392, and in‐source dimer [2 M+ H^+^] at 783, and some minor impurities of Zwittergent 3–12 seen as MH^+^ at 336. The peptide samples were loaded to a 0.25 mL C8 OptiPak trapping cartridge custom‐packed with Michrom Magic (Optimize Technologies) C8, washed, then switched in‐line with a 20 cm by 75 mmC18 packed spray tip nano column packed with Michrom Magic C18AQ, for a 2‐step gradient. Mobile phase A was water/acetonitrile/formic acid (98/2/0.2) and mobile phase B was acetonitrile/isopropanol/water/formic acid (80/10/10/0.2). Using a flow rate of 350 nL/min, a 90 min, 2‐step LC gradient was run from 5% B to 50% B in 60 min, followed by 50%–95% B over the next 10 min, hold 10 min at 95% B, back to starting conditions and re‐equilibrated.

### 4.9 LC–MS/MS analysis

Electrospray tandem mass spectrometry (LC–MS/MS) was performed at the Mayo Clinic Proteomics Core on a Thermo Q‐Exactive Orbitrap mass spectrometer, using a 70 000 RP survey scan in profile mode, m/z 340–2000 Da, with lock masses, followed by 20 MSMS HCD fragmentation scans at 17500 resolution on doubly and triply charged precursors. Single charged ions were excluded, and ions selected for MS/MS were placed on an exclusion list for 60 s.

### 4.10 LC–MS/MS data analysis, statistical analysis

All LC–MS/MS *.raw Data files were analyzed with MaxQuant version 1.5.2.8, searching against the SPROT *S. cerevisiae* database downloaded September 28, 2017 and searched using the following criteria: LFQ quantification with a min of 1 high confidence peptide. Trypsin was selected as the protease with max miss cleavage set to 2. Carbamidomethyl (C) was selected as a fixed modification. Variable modifications were set to Deamidation (NQ), Oxidization (M), Formylation (n‐term) and Phosphorylation (STY). Orbitrap mass spectrometer was selected using a MS error of 20 ppm and a MS/MS error of 0.5 Da. A 1% FDR cutoff was selected for peptide, protein, and site identifications. LFQ Intensities were reported based on the MS level peak areas determined by MaxQuant and reported in the proteinGroups.txt file. Proteins were removed from the results file if they were flagged by MaxQuant as “Contaminants,” “Reverse” or “Only identified by site.” Complete three biological replicates were performed. The abundance data from each biological replicate were normalized to the ratio of Vps501 bait protein in that biological replicate. LFQ Peak intensities were analyzed in each run to determine protein hits that fell into the category of either Vps501 elution (VE) only hits or Bead elution (BE) only hits and retained if they confirmed to VE state across all three runs. LFQ Sig cutoffs are Sig Up >1.2 ratio (Log2 0.26) and Sig Down <0.8 ratio (Log2‐0.32). Any hits that were not observed in at least two replicates each were labeled “no quant” (a normalized ratio was still calculated but not included in final data set analysis). A list of proteins identified and corresponding ratios can be found in Supplemental Materials. The mass spectrometry proteomic data have been deposited to the ProteomeXchange Consortium (http://proteomecentral.proteomexchange.org)^67^ via the PRIDE partner repository.^68^, ^69^

### 4.11 Vps501 dimer purification and liposome binding assay

His‐tag fusion vector of Vps501 was made by synthesizing the Vps501 ORF as a NdeI and HindIII fragment (Genscript). The gene synthesis product was cloned in‐frame into NdeI and HindIII sites of vector pET‐30a(+) (Novagen), and the resulting plasmid was sequenced. The plasmid was transformed into *E. coli* BL21 and expressed by IPTG. Briefly, plasmids were transformed into BL21(DE3) competent cells and grown in LB containing the appropriate antibiotics in baffled flasks filled to 20% of the total volume. IPTG solution was added to a final concentration of 0.1 mM and cultures were grown at 37°C for 4 h, then shifted to RT for 20 h. Recombinant Vps501 contained a C‐terminal His‐tag and was purified using an ÄKTA START fast protein liquid chromatography system (Cytivia) equipped with a 1 mL His‐Trap HP column followed by buffer exchange into assay buffer. 6XHIS‐Vps501^YKAA^, was created by site‐directed mutagenesis and purified as described above with an additional step of size‐exclusion chromatography (HiPREP16/60 Sephacryl S‐300 HR, Cytivia) to remove additional impurities. Snx4‐Atg20 were purified as previously described.^70^ All purified protein concentrations were quantified by Brafford assay (Pierce).

Liposome binding assays were carried out as previously described.^6^ Briefly, to test binding to PI containing liposomes, liposome compositions were (2.5 mM lipid) 0% or 11% PtdIns(3)P; 0% or 18% PtdIns(3,5)P2; with PC adjusted to 100%. To test PS binding, liposomes (0% or 30% PS; 1% PtdIns(3)P; 20% ergosterol; with PC adjusted to 100%) were incubated with 4 μM Snx4‐Atg20, Vps501‐Vps501 or Vps501^YKAA^ ‐Vps501^YKAA^ for 30 min at 30°C and sedimented at 100000 x g for 20 min. Pellet (P) and supernatant (S) fractions were loaded onto 10% polyacrylamide gels and visualized using Coomassie Brilliant Blue stain. Band intensities were quantified using Quantity One 1D analysis software (Bio‐rad) and proportion of SNX‐BAR proteins in pellet fraction was quantified. Two‐way ANOVA was used to determine statistical significance (GraphPad Prism 7.01).

## CONFLICT OF INTEREST

The authors have no conflict of interest to declare.

**Abbreviations List**
BAR: Bin‐Amphiphysin‐Rvs161 (BAR) homology
CBB: coomassie brilliant blue
GFP: Green Fluorescent Protein
ORF: open reading frame
PGK: phosphoglycerate kinase
PX: Phox homology domain
PI3P: phosphatidylinositol‐3‐phosphate
ROI: region of interest
SNX: sorting nexin
SNX‐BAR: sorting nexin containing a BAR domain
TEN: tubular endosomal network
VL: vacuolar lumen
VM: vacuolar membrane
WT: wild‐type

5

### PEER REVIEW

The peer review history for this article is available at https://publons.com/publon/10.1111/tra.12833.

## Supporting information


Table S1

**Figure S1** Sequence alignment of YKR078W/VPS501 to Vps5 proteins from other yeast taxa. Full‐length sequences for all taxa for each gene family were aligned with Muscle 3.6^58^ and edited manually in the case of clear errors. Maximum likelihood analyses were conducted with RAxML v.8.2.4^59^ using a LG + G matrix model determined by ProtTest v.3^60^ and a trimmed alignment containing the conserved PX‐BAR domains. Sc, *S. cerevisiae*, Cg, *C. glabrata*, Eg, *E. gossypi*, Sp, *S. pombe*.

**Figure S2** Vps501 interactions with the SEACAT complex during autophagy. (A) Maximum projection micrographs of cells expressing GFP‐Atg8 in wildtype and indicated mutant cells before and after nitrogen starvation. The scale bar indicates 5 μm. (B) Quantitative immunoblotting was used to assess GFP‐Atg8 flux before and after autophagy induction. A partial reduction in GFP‐Atg8 flux is seen when Vps501 is ablated in combination with each of the SEACAT subunits with the most significant defect occurring in *seh1Δvps501Δ* cells. A representative immunoblot is shown. Anti‐Pgk1 was used as a loading control. (C) Graph of quantification of GFP‐Atg8 processing. The results are from three experiments and averaged using the standard error of the mean. Indicated significance is a comparison of wildtype to single deletions or double mutants. *p < 0.05, ** p < 0.01, ***p < 0.001 indicates significance as calculated by Student's t‐test.

**Figure S3** Autophagy influx is defective in *vps501*Δ*sea1*Δ cells during rapamycin treatment.
(A) Maximum projection micrographs of cells expressing GFP‐Atg8 in wildtype and indicated mutant cells before and after rapamycin treatment. The scale bar indicates 5 μm. (B) Quantitative immunoblotting was used to assess GFP‐Atg8 flux before and after autophagy induction by rapamycin. A significant reduction in GFP‐Atg8 flux is seen when Vps501 is ablated in combination with Sea1. A representative immunoblot is shown. Anti‐Pgk1 was used as a loading control. (C) Graph of quantification of GFP‐Atg8 processing. The results are from three experiments and averaged using the standard error of the mean. Indicated significance is a comparison of wildtype to single deletions or double mutants. *p < 0.05, ** p < 0.01, ***p < 0.001 indicates significance as calculated by Student's t‐test.

**Figure S4** SEACAT subunits, Sea2‐GFP, Sea3‐GFP, Sea4‐GFP, Seh1‐GFP and Sec13‐GFP are partially mislocalized in *vps501*Δ*sea1*Δ cells. (A) Sea2‐GFP, Sea3‐GFP, Sea4‐GFP and Sea1‐GFP reside on the vacuolar membrane in wildtype and *vps501*Δ cells, but localize to the vacuolar lumen (VL) in *sea1*Δ cells and *vps501*Δ*sea1*Δ cells. *sea1*Δ cells appear to mask any effects of VPS501. (B) VL localization is defined by visually scoring the presence of GFP in the VL. C) SEACAT subunits, Seh1‐GFP and Sec13‐GFP localize to the vacuolar membrane in wildtype and *vps501*Δ cells, but are enriched in non‐vacuolar compartments in *sea1*Δ cells and *vps501*Δ*sea1*Δ cells. Seh1 and Sec13 have previously‐reported nuclear and ER roles, respectively, and are likely enriched on these structures when vacuolar membrane localization is compromised. (B) VL localization as determined by calculating the percentage of cells with GFP signal on the vacuole using CMAC as a visual maker to determine vacuole boundaries. The results are from three experiments and averaged using the standard error of the mean. Indicated significance is a comparison of wildtype to single deletions or double mutants. *p < 0.05, ** p < 0.01, ***p < 0.001 indicates significance as calculated by Student's t‐test.

**Figure S5** Other vacuolar membrane proteins are mislocalized in *vps501*Δ*sea1*Δ cells. (A) Micrographs of Atg27‐2XGFP in wildtype and indicated mutant cells. Atg27‐2XGFP is typically localized to the vacuolar membrane, the Golgi and endosomal compartments. In *atg1*Δ cells, Atg27‐2XGFP cycling is less abundant on the vacuolar membrane. In *vps501∆sea1∆* cells, Atg27‐2XGFP is significantly depleted from the vacuolar membrane, indicating Atg27 cycling to and from the vacuolar membrane is dependent on Vps501 and Sea1 function during autophagy induction. (B) Graph of the quantification of Atg27‐2XGFP vacuolar localization as described in the text. Percentage of cells with Atg27 vacuolar localization is between 85–95% in wildtype cells or in cells ablated for only Vps501 or Sea1 and is reduced ~20% *atg1*Δ cells and ~ 75% in *vps501*Δ*sea1*Δ cells. The results are from three experiments and averaged using the standard error of the mean. Indicated significance is a comparison of wildtype to single deletions or double mutants. *p < 0.05, ** p < 0.01, ***p < 0.001 indicates significance as calculated by Student's *t*‐test. The scale bar indicates 5 μm.

## Data Availability

The authors confirm that the data supporting the findings of this study are available within the article [and/or] its supplementary materials.
