# Supplementary figures and images for: Vps501, a novel vacuolar SNX‐BAR protein cooperates with the SEA complex to regulate TORC1 signaling
Source: Traffic. 2022 Feb 15;23(4):192–207. doi: 10.1111/tra.12833 (PMC9305297; doi:10.1111/tra.12833)

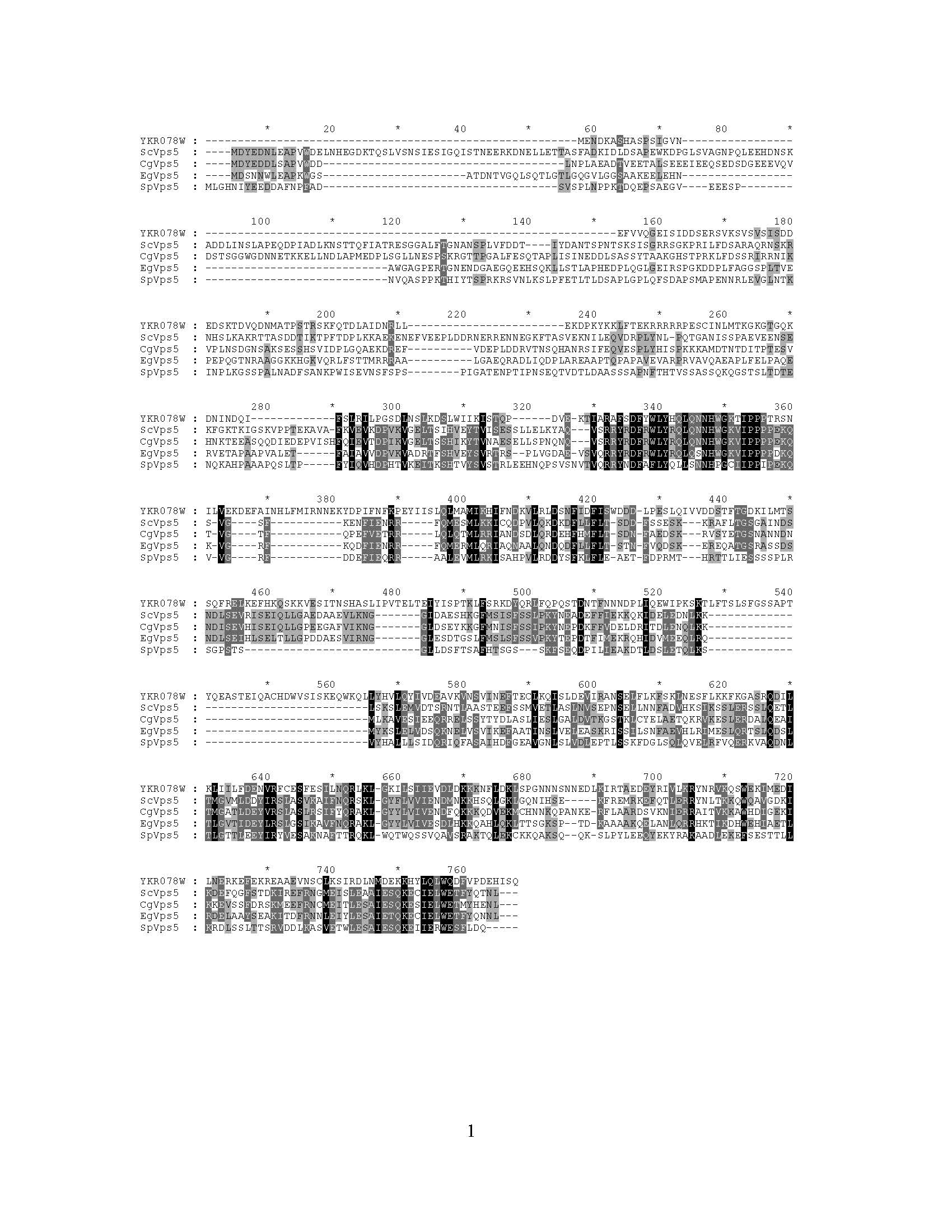

Supplement: Supplementary file 2 — Figure S1 Sequence alignment of YKR078W/VPS501 to Vps5 proteins from other yeast taxa. Full‐length sequences for all taxa for each gene family were aligned with Muscle 3.6 58 and edited manually in the case of clear errors. Maximum likelihood analyses were conducted with RAxML v.8.2.4 59 using a LG + G matrix model determined by ProtTest v.3 60 and a trimmed alignment containing the conserved PX‐BAR domains. Sc, S. cerevisiae, Cg, C. glabrata, Eg, E. gossypi, Sp, S. pombe. [file TRA-23-192-s004.jpg]

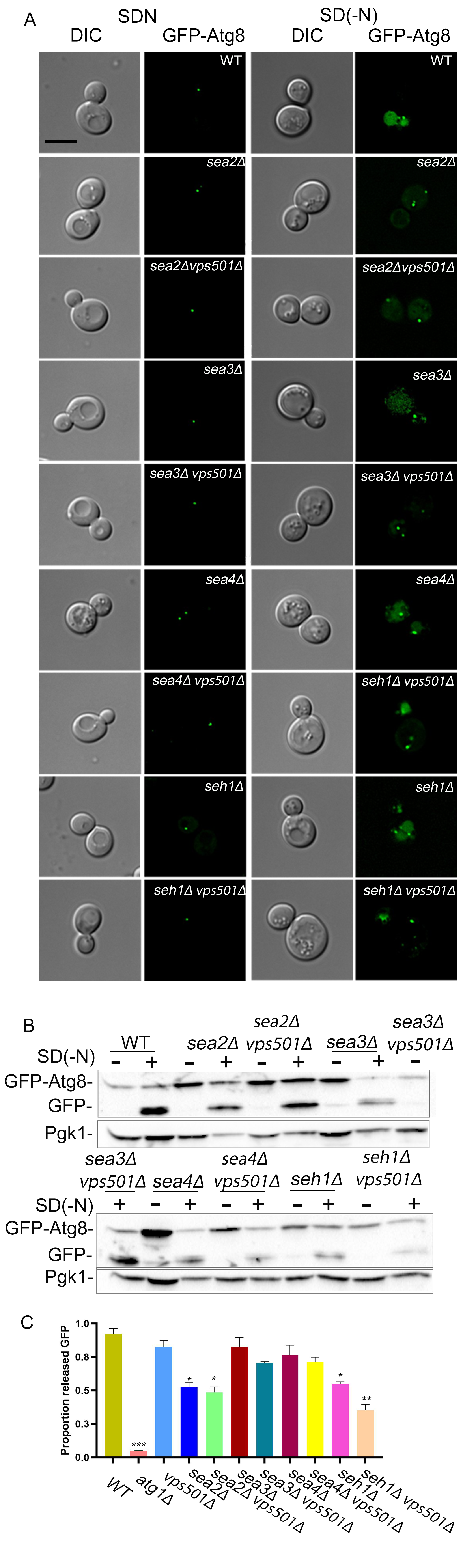

Supplement: Supplementary file 3 — Figure S2 Vps501 interactions with the SEACAT complex during autophagy. (A) Maximum projection micrographs of cells expressing GFP‐Atg8 in wildtype and indicated mutant cells before and after nitrogen starvation. The scale bar indicates 5 μm. (B) Quantitative immunoblotting was used to assess GFP‐Atg8 flux before and after autophagy induction. A partial reduction in GFP‐Atg8 flux is seen when Vps501 is ablated in combination with each of the SEACAT subunits with the most significant defect occurring in seh1Δvps501Δ cells. A representative immunoblot is shown. Anti‐Pgk1 was used as a loading control. (C) Graph of quantification of GFP‐Atg8 processing. The results are from three experiments and averaged using the standard error of the mean. Indicated significance is a comparison of wildtype to single deletions or double mutants. *p < 0.05, ** p < 0.01, ***p < 0.001 indicates significance as calculated by Student's t‐test. [file TRA-23-192-s005.tif]

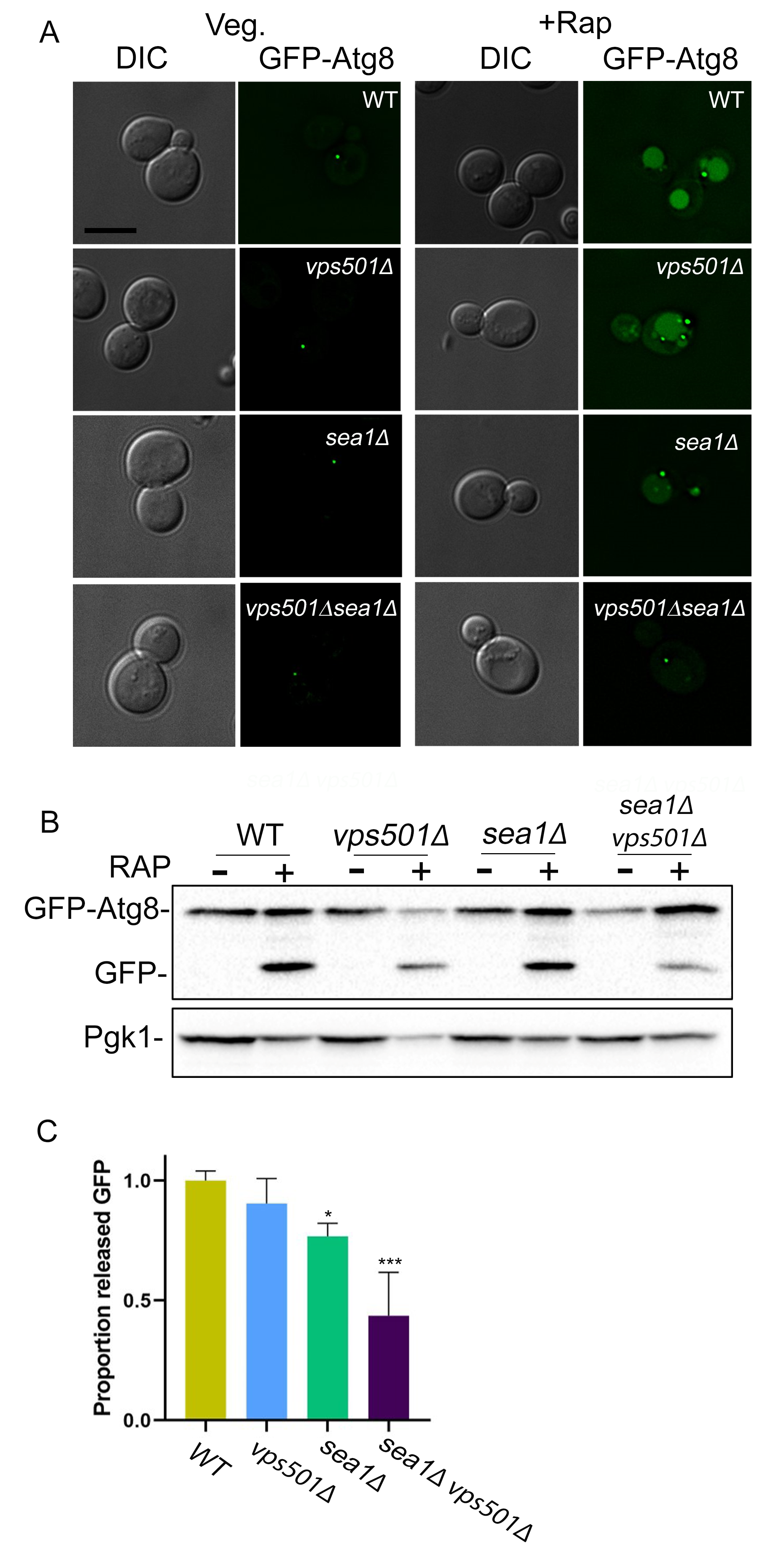

Supplement: Supplementary file 4 — Figure S3 Autophagy influx is defective in vps501Δsea1Δ cells during rapamycin treatment. (A) Maximum projection micrographs of cells expressing GFP‐Atg8 in wildtype and indicated mutant cells before and after rapamycin treatment. The scale bar indicates 5 μm. (B) Quantitative immunoblotting was used to assess GFP‐Atg8 flux before and after autophagy induction by rapamycin. A significant reduction in GFP‐Atg8 flux is seen when Vps501 is ablated in combination with Sea1. A representative immunoblot is shown. Anti‐Pgk1 was used as a loading control. (C) Graph of quantification of GFP‐Atg8 processing. The results are from three experiments and averaged using the standard error of the mean. Indicated significance is a comparison of wildtype to single deletions or double mutants. *p < 0.05, ** p < 0.01, ***p < 0.001 indicates significance as calculated by Student's t‐test. [file TRA-23-192-s002.tif]

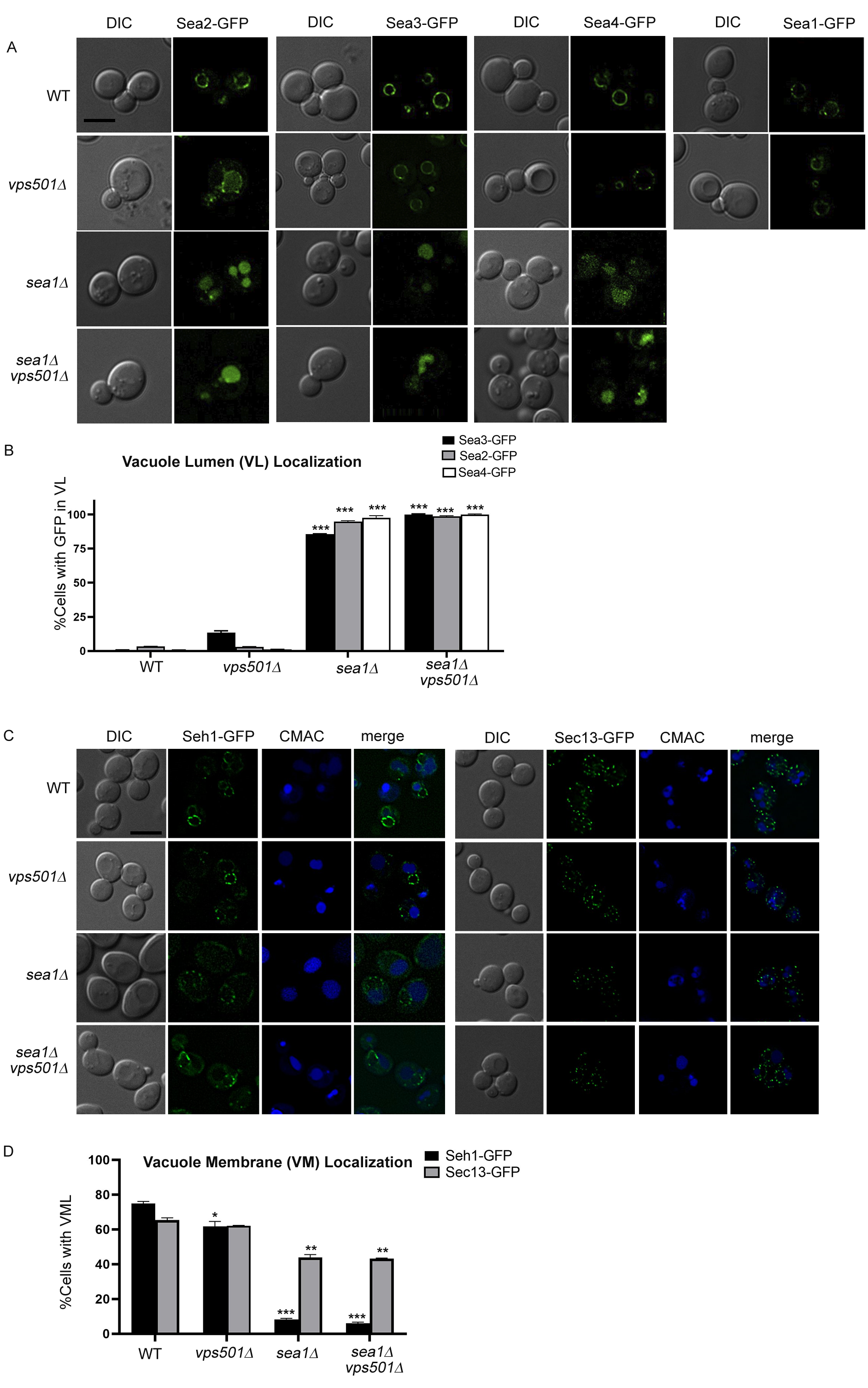

Supplement: Supplementary file 5 — Figure S4 SEACAT subunits, Sea2‐GFP, Sea3‐GFP, Sea4‐GFP, Seh1‐GFP and Sec13‐GFP are partially mislocalized in vps501Δsea1Δ cells. (A) Sea2‐GFP, Sea3‐GFP, Sea4‐GFP and Sea1‐GFP reside on the vacuolar membrane in wildtype and vps501Δ cells, but localize to the vacuolar lumen (VL) in sea1Δ cells and vps501Δsea1Δ cells. sea1Δ cells appear to mask any effects of VPS501. (B) VL localization is defined by visually scoring the presence of GFP in the VL. C) SEACAT subunits, Seh1‐GFP and Sec13‐GFP localize to the vacuolar membrane in wildtype and vps501Δ cells, but are enriched in non‐vacuolar compartments in sea1Δ cells and vps501Δsea1Δ cells. Seh1 and Sec13 have previously‐reported nuclear and ER roles, respectively, and are likely enriched on these structures when vacuolar membrane localization is compromised. (B) VL localization as determined by calculating the percentage of cells with GFP signal on the vacuole using CMAC as a visual maker to determine vacuole boundaries. The results are from three experiments and averaged using the standard error of the mean. Indicated significance is a comparison of wildtype to single deletions or double mutants. *p < 0.05, ** p < 0.01, ***p < 0.001 indicates significance as calculated by Student's t‐test. [file TRA-23-192-s003.tif]

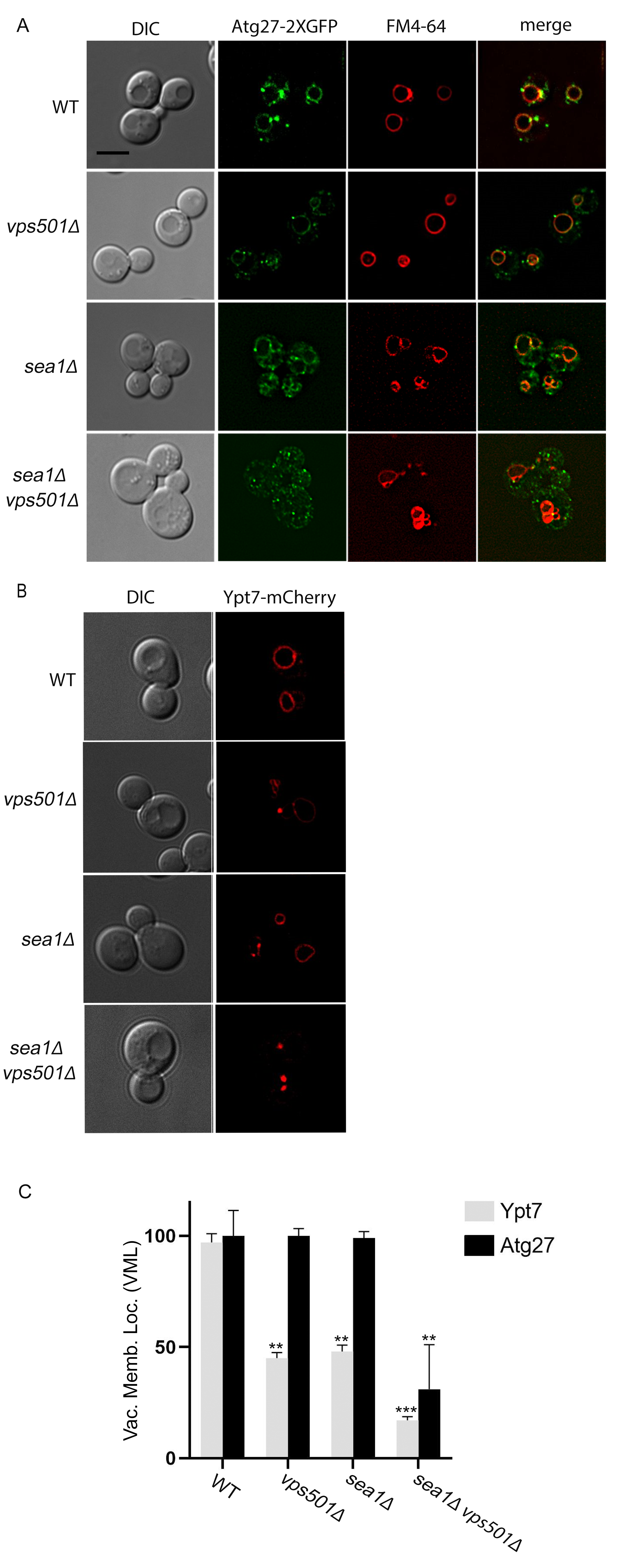

Supplement: Supplementary file 6 — Figure S5 Other vacuolar membrane proteins are mislocalized in vps501Δsea1Δ cells. (A) Micrographs of Atg27‐2XGFP in wildtype and indicated mutant cells. Atg27‐2XGFP is typically localized to the vacuolar membrane, the Golgi and endosomal compartments. In atg1Δ cells, Atg27‐2XGFP cycling is less abundant on the vacuolar membrane. In vps501∆sea1∆ cells, Atg27‐2XGFP is significantly depleted from the vacuolar membrane, indicating Atg27 cycling to and from the vacuolar membrane is dependent on Vps501 and Sea1 function during autophagy induction. (B) Graph of the quantification of Atg27‐2XGFP vacuolar localization as described in the text. Percentage of cells with Atg27 vacuolar localization is between 85–95% in wildtype cells or in cells ablated for only Vps501 or Sea1 and is reduced ~20% atg1Δ cells and ~ 75% in vps501Δsea1Δ cells. The results are from three experiments and averaged using the standard error of the mean. Indicated significance is a comparison of wildtype to single deletions or double mutants. *p < 0.05, ** p < 0.01, ***p < 0.001 indicates significance as calculated by Student's t‐test. The scale bar indicates 5 μm. [file TRA-23-192-s006.tif]
